# Multicenter phase II trial of trastuzumab and docetaxel for HER2-positive salivary gland cancer

**DOI:** 10.1093/jjco/hyaf106

**Published:** 2025-06-25

**Authors:** Satoshi Kano, Naomi Kiyota, Ichiro Kinoshita, Yuichiro Tada, Kei Ijichi, Tomoko Yamazaki, Yasushi Shimizu, Yutaka Hatanaka, Hitoshi Tsuda, Shojiroh Morinaga, Yoshihiro Matsuno, Yoichi M Ito, Naoki Nishimoto, Keiko Kobayashi, Toshiyuki Isoe, Takuro Noguchi, Akihiro Homma, Hirotoshi Dosaka-Akita

**Affiliations:** Department of Otolaryngology-Head and Neck Surgery, Faculty of Medicine and Graduate School of Medicine, Hokkaido University, N15, W7, Kita-ku, Sapporo, 060-8638, Japan; Department of Medical Oncology and Hematology, Kobe University Hospital, 7-5-2 Kusunoki-ku, Kobe, 650-0017, Japan; Kobe University Hospital Cancer Center, 7-5-2 Kusunoki-ku, Kobe, 650-0017, Japan; Department of Medical Oncology, Hokkaido University Hospital, N14, W5, Kita-ku, Sapporo, 060-8648, Japan; Department of Head and Neck Oncology and Surgery, International University of Health and Welfare, Mita Hospital, 1-4-3 Mita, Minato-ku, Tokyo, 108-8329, Japan; Department of Otolaryngology- Head and Neck Surgery, Nagoya City University Graduate School of Medicine, 1 Kawasumi, Mizuhomachi, Mizuho-ku, Nagoya, 467-8601, Japan; Division of Head and Neck Cancer Oncology, Miyagi Cancer Center, 47-1 Nodayama, Aza-Shiote, Aijima, Natori, 981-1293, Japan; Department of Medical Oncology, Hokkaido University Hospital, N14, W5, Kita-ku, Sapporo, 060-8648, Japan; Center for Development of Advanced Diagnostics, Hokkaido University Hospital, N14, W5, Kita-ku, Sapporo, 060-8648, Japan; Department of Basic Pathology, National Defense Medical College, 3-2 Namiki, Tokorozawa, 359-8513, Japan; Department of Diagnostic Pathology, Hino Municipal Hospital, 3-1, 4-chome, Tamadaira, Hino, 191-0062, Japan; Department of Surgical Pathology, Hokkaido University Hospital, N14, W5, Kita-ku, Sapporo, 060-8648, Japan; Data Science Center, Promotion Unit, Institute of Health Science Innovation for Medical Care, Hokkaido University Hospital, N14, W5, Kita-ku, Sapporo, 060-8648, Japan; Clinical Research and Medical Innovation Center, Hokkaido University Hospital, N14, W5, Kita-ku, Sapporo, 060-8648, Japan; Clinical Research and Medical Innovation Center, Hokkaido University Hospital, N14, W5, Kita-ku, Sapporo, 060-8648, Japan; Clinical Research and Medical Innovation Center, Hokkaido University Hospital, N14, W5, Kita-ku, Sapporo, 060-8648, Japan; Department of Medical Oncology, Hokkaido University Hospital, N14, W5, Kita-ku, Sapporo, 060-8648, Japan; Department of Otolaryngology-Head and Neck Surgery, Faculty of Medicine and Graduate School of Medicine, Hokkaido University, N15, W7, Kita-ku, Sapporo, 060-8638, Japan; Department of Medical Oncology, Hokkaido University Hospital, N14, W5, Kita-ku, Sapporo, 060-8648, Japan

**Keywords:** trastuzumab, docetaxel, HER2, phase II trial, salivary gland cancer

## Abstract

**Backgrounds:**

Standard systemic chemotherapy remains unestablished for recurrent or metastatic (RM) salivary gland cancer (SGC) due to its rarity. A single institute phase II trial of trastuzumab and docetaxel previously showed efficacy with human epidermal growth factor receptor 2 (HER2)-positive SGC.

**Methods:**

We conducted a multicenter, single-arm, open-label phase II trial of trastuzumab and docetaxel for HER2-positive RM SGC. Patients received trastuzumab 6 mg/kg (loading dose 8 mg/kg) and docetaxel 70 mg/m^2^ every 3 weeks up to eight cycles. The primary endpoint was the objective response rate (ORR) by a blinded independent review committee. Secondary endpoints included progression-free survival (PFS), overall survival (OS), disease control rate (DCR), and safety. Forty-eight patients were screened for HER2 status; 23 were HER2-positive.

**Results:**

Eighteen patients were enrolled, with 16 receiving the protocol treatment. Fourteen patients were diagnosed with salivary duct carcinoma. The ORR was 60.0% (95% confidence interval [CI], 32.3 to 83.7). The median PFS was 8.5 months (95% CI, 6.0 to 12.7), the median OS, 33.8 months (95% CI, 16.9 to not estimable), and the DCR, 93.3% (95% CI, 68.1 to 99.8). The most frequent grade ≥3 treatment-emerged adverse events were neutropenia (100%), leukopenia (93.8%), lymphopenia (18.8%), and febrile neutropenia (12.5%). One treatment-related death occurred (6.3%) due to hypoalbuminemia.

**Conclusions:**

These results demonstrate the significant efficacy and predictable toxicities of trastuzumab and docetaxel in patients with HER2-positive RM SGC, leading to the simultaneous approval of trastuzumab and HER2 companion diagnostics assay for this setting in Japan.

## Teaser

Trastuzumab plus docetaxel showed significant efficacy and manageable toxicity in HER2-positive recurrent/metastatic salivary gland cancer, leading to simultaneous approval in Japan with HER2 companion diagnostics.

## Introduction

Salivary gland cancer (SGC) is a rare neoplasm, representing ~0.3% of all malignancies and 8% of head and neck cancers [1,2]. In 2020, the number of new cases and deaths associated with SGC worldwide accounted for ~54 000 and 23 000, respectively [2]. While surgical resection and radiotherapy for curative intent are recommended for local disease [1], systemic therapy for recurrent or metastatic (RM) disease remains unestablished [3,4]. Small phase II studies of SGC using various chemotherapeutic drugs, primarily platinum and taxane, have demonstrated limited efficacy, with objective response rates (ORRs) ranging from 10% to 35%, mostly less than 30%, [5–12] while subset and retrospective studies of SGC from head and neck cancer have shown higher ORR of 39% to 60% [13–18].

Human epidermal growth factor receptor 2 (HER2) overexpression varies across different histologic types of SGC, with the highest prevalence in salivary duct carcinoma (SDC) (43.0%), followed by carcinoma ex pleomorphic adenoma (39.0%) [19]. The HER2 overexpression is commonly correlated with aggressive histological subtypes and poorer prognosis [20]. Although monotherapy with the anti-HER2 antibody trastuzumab demonstrated a modest ORR of 7% in a phase II trial of HER2-positive (immunohistochemistry [IHC] 2+ or 3+) SGC [21], several case reports have described notable responses to trastuzumab combined with taxanes [22,23]. Subsequently, a single-institution phase II trial of trastuzumab plus docetaxel reported ORR of 70.2% in 57 patients with HER2-positive (IHC 3+ or gene amplification) SDC [24].

These results underscore the benefits of trastuzumab-based therapy, including trastuzumab and docetaxel for treating advanced SGC. However, HER2-targeted therapy was not approved for SGC based on the above results. We thus conducted a multicenter phase II clinical trial to investigate the efficacy and safety of trastuzumab and docetaxel in patients with HER2-positive SGC, pursuant to the Japanese good clinical practice (J-GCP) with the aim of regulatory approval.

## Materials and methods

### Patients and study design

This was a multicenter, single-arm, open-label phase II trial in Japan conducted as an investigator-initiated study. Eligible patients were aged 20 to 75 years, histologically diagnosed with SGC, and presented with RM disease incurable by radical treatment. They had measurable lesions according to RECIST v1.1 and an Eastern Cooperative Oncology Group (ECOG) performance status of 0 to 2. Patients were required to have an expected survival of at least 3 months and to provide written informed consent. Key laboratory values within 14 days before registration included a neutrophil count ≥1500/mm^3^, hemoglobin ≥8.0 g/dl, platelet count ≥10 × 10 [4]/mm^3^, total bilirubin ≤1.5 mg/dl, AST and ALT ≤2.5 × upper limit of normal (ULN), serum creatinine ≤2.0 × ULN, and SpO_2_ ≥ 90%. Exclusion criteria included previous use of taxane-based anticancer agents or trastuzumab, recent anticancer therapies within 4 weeks, significant cardiovascular conditions, a left ventricular ejection fraction (LVEF) <50%, and severe comorbidities. Patients with interstitial pneumonia, pulmonary fibrosis, or other advanced malignancies causing dyspnea or requiring oxygen therapy were excluded. Those with active infections, severe drug allergies, or requiring systemic corticosteroids or immunosuppressive therapy were ineligible. The full inclusion and exclusion criteria were described in the supporting information (Supplementary S1).

To evaluate HER2 protein overexpression and gene amplification, we subjected the paraffin specimens from the primary tumor or metastatic lesions to IHC using Ventana ultraView Pathway HER2 (4B5) (Roche Diagnostics, Mannheim, Germany) and dual in situ hybridization (DISH) using Ventana Inform Dual ISH HER2 Kit (Roche Diagnostics) at the central assessment laboratory (Department of Pathology, Hokkaido University Hospital), according to the manufacturer’s instructions. Results indicating IHC 3+, or IHC 2+ and DISH positivity (HER2/CEP17 ratio ≥ 2.0) were considered HER2-positive. All tumors were centrally reviewed for histopathology and HER2 status by YM, HT, and SM.

This trial protocol was approved by the institutional review board of each participating institution (Hokkaido University Hospital, International University of Health and Welfare Mita Hospital, Nagoya City University Hospital, Kobe University Hospital, and Miyagi Cancer Center). All patients were required to provide written informed consent before enrollment. This trial was registered under the University Medical Hospital Information Network Clinical Trials Registry (UMIN-CTR); Identifier UMIN000018165 (the date of the first registration: 6 July 6 2017). The trial was performed in accordance with the Declaration of Helsinki and the J-GCP.

### Treatment

Eligible patients received 8 mg/kg of trastuzumab for the initial dose and 6 mg/kg for the second and subsequent doses every 3 weeks (Supplementary S2). Docetaxel was administered at 70 mg/m2 every 3 weeks. All subjects were to receive the protocol treatment combining trastuzumab and docetaxel for a maximum of eight cycles. This trial referenced the dosage and number of cycles from the prior phase II trial [24]. Treatment delay, infusion interruption, and discontinuation were performed according to the criteria of the protocol (Supplementary S3 and S4). The docetaxel dosage reduction was conducted in two steps, first to 55 mg/m^2^ and then to 45 mg/m^2^, in line with the criteria (Supplementary S3, section 2.3).

Protocol treatment was discontinued before completing eight cycles in cases of disease progression, the development of unacceptable adverse events (AEs), or patient withdrawal (Supplementary S4). Subsequent therapy after the protocol treatment was not predefined.

Premedication with corticosteroids was permitted at the discretion of the investigator or sub-investigator to mitigate the prevalence and severity of fluid retention by docetaxel (Supplementary S3, section 2.6). Bisphosphonates, denosumab, and prophylactic and therapeutic use of granulocyte colony-stimulating factor (G-CSF) agents according to insurance coverage were allowed (Supplementary S5). Other medications, including antibiotics, for the treatment or prevention of AEs were also allowed.

### Assessment

The primary endpoint was the ORR. Secondary endpoints were progression-free survival (PFS), overall survival (OS), disease control rate (DCR), and safety. Imaging assessments were executed every 6 weeks. Anti-tumor efficacy was evaluated in accordance with RECIST v1.1 criteria by a blinded independent review committee (BIRC). ORR and DCR were delineated as the proportion of patients who had either a complete response (CR) or a partial response (PR), and CR, PR, or stable disease (SD) in the best objective response by RECIST v1.1, respectively. If a patient was documented as having CR or PR, confirmatory evaluation was performed after an interval of at least 4 weeks. SD required a minimum 6-week period from enrolment in the trial.

PFS was defined as the time from the date of the first administration to one of the following events: progressive disease (PD) by RECIST v1.1, clinical deterioration of primary disease, and the appearance of new lesions not confirmed by imaging (clinical progression), or death. If none of these events occurred, and the patient was alive without progression at the time of the last follow-up, PFS was censored at that time. If subsequent treatment was initiated in patients without progression of the protocol treatment, PFS was censored at the date when the absence of progression was confirmed before subsequent treatment. Maintenance therapy of trastuzumab plus docetaxel, or either drug, was considered subsequent therapy. PFS was determined based on the assessment results of BIRC.

OS was defined as the time from the date of the first administration of the investigational drug to the date of death from any cause. For patients who were alive at the time of the last follow-up, OS was censored at that date. For patients lost to follow-up, OS was censored at the last date on which survival was confirmed.

Time to response (TTR) was defined as the duration from the first administration to the first documented partial or CR, and was assessed only in patients who achieved a confirmed CR or PR.

Duration of response (DoR) was defined for patients with confirmed CR or PR as the time from the date of first documented partial or CR to one of the following events: PD by RECIST v1.1, clinical deterioration of primary disease and the appearance of new lesions not confirmed by imaging (clinical progression), or death. The definition of censoring for DoR was the same as that for PFS.

Duration of SD was defined from the date of the first administration to one of the following events: PD by RECIST v1.1, clinical deterioration of primary disease and the appearance of new lesions not confirmed by imaging (clinical progression), or death, for patients who achieved SD. The definition of censoring for duration of SD was the same as that for PFS.

AEs were evaluated using the Japanese translation of common terminology criteria for adverse events (CTCAE) v4.0 Japan Clinical Oncology Group (JCOG) version (CTCAE v4.03/medical dictionary for regulatory activities [MedDRA] v12.0), listing AE items and determining their grades. In the aggregation of AEs, the AEs recorded in the case report forms were coded and classified by system organ class (SOC) using MedDRA/J version 22.0 and were presented by preferred term (PT). Electrocardiograms and echocardiography or multigated acquisition were performed every four cycles.

Treatment-emergent adverse events (TEAEs) were defined as AEs that occurred after the protocol treatment initiation or worsened relative to the pretreatment state in this trial. The investigator assessed the seriousness, severity, and relationship of AEs to the trial drugs. Treatment-related adverse events (TRAEs) were those for which a causal relationship with the protocol treatment cannot be ruled out. Serious adverse events (SAEs) were defined as AEs that met any of the following criteria: death, life-threatening, required hospitalization or prolongation of existing hospitalization for treatment, disability, potential to result in disability, other events deemed serious by the investigator in accordance with the above criteria, and congenital anomaly or birth defect in subsequent generations. The relative dose intensity (RDI) was calculated as the ratio of the actual to the planned dose intensity.

Subjects who received at least one dose of trastuzumab and met the inclusion criteria without violating any exclusion criteria were defined as the full analysis set (FAS). Efficacy analysis was conducted on the FAS. Subjects who received at least one dose of trastuzumab were defined as the safety analysis set (SAS). Safety analysis was performed on the SAS.

### Statistical analysis

The first data cutoff date was set at the end of the first survey of the prognosis of the last enrolled case (the completion of protocol treatment or 6 months after discontinuation). Interim analyses were performed using this fixed data. After the clinical trial period’s termination, the data were fixed again, and the final analysis was executed. The data update from the interim analysis was limited to PFS and OS and changes in the coding of AEs due to MedDRA version updates.

The duration of OS and PFS was estimated using the Kaplan–Meier method, and their 95% confidence intervals (CIs) were approximated using the Brookmeyer and Crowley method. Clopper–Pearson 95% CIs were calculated for the ORR and the DCR.

For sample size calculation, a threshold ORR was set at 25%, based on the clinical trials and retrospective studies available at the start of this trial [10,12,17,18], which is still relevant given the phase II trials of chemotherapy for SGC reported until now [5–12]. The expected ORR was set at 70% based on the ORR in the interim report of the single institution trial (Tada Y, personal communication); the final analysis, conducted later, showed an ORR of 70.2% [24]. Assuming a two-sided alpha error of 0.05 (significance level of 5%), the minimum number of patients required to achieve a power of 90% or more for the lower limit of the Clopper–Pearson exact 95% CI to exceed the threshold would be 14 cases. The target number of patients was set at 16, assuming a dropout of two patients.

## Results

### Patients

**Figure 1:**
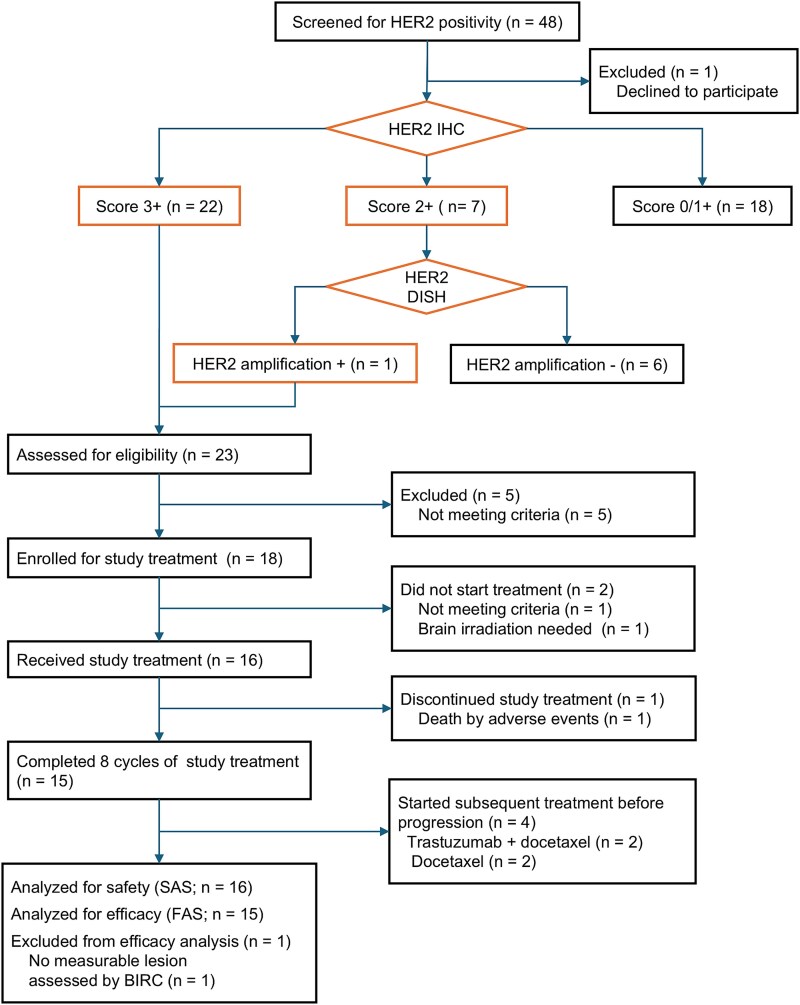
Trial profile. Abbreviations: SAS, safety analysis set. FAS, full analysis set. BIRC, BIRC.

Between July 2015 and April 2017, 48 patients were screened for HER2 positivity in this trial, and 23 patients were assessed as HER2-positive; 22 were HER2 IHC 3+, and one was HER2 IHC 2+ with DISH + (Fig. 1). After excluding patients who did not meet the eligibility criteria, 18 were enrolled between 8 August 2015 and 25 April 2017. One patient was found not to meet eligibility criteria, and another was referred to radiotherapy for brain metastasis after enrollment. Consequently, 16 patients received the protocol treatment. The first and the second (final) data cutoffs were 2 April 2018 and 12 March 2019, respectively. Table 1 summarizes the baseline characteristics of the patients. The median age was 59 years (range 26–72), with a predominantly male demographic (81%). Primary sites included parotid glands in 69% of cases and submandibular glands in 25%. Central pathological assessment diagnosed SDC in most patients (88%). HER2 IHC3+ was ascertained in all patients. Most patients had previously undergone surgery, chemoradiotherapy, or radiotherapy before enrollment.

**Table 1:** Patient characteristics

	**N = 16** ^**a**^
Characteristics	Number of patients (%)^**b**^
Age (year)	median (range): 59 (26–72)
Gender	
Male	13 (81.3)
Female	3 (18.8)
ECOG PS	
0	11 (68.8)
1	5 (31.3)
2	0 (0.0)
Primary site	
Parotid gland	11 (68.8)
Submandibular gland	4 (25.0)
Others	1 (6.3)
Pathology central diagnosis	
SDC	14 (87.5)
Carcinoma compatible with SDC	2 (12.5)
HER2 status	
IHC 3+	16 (100)
Previous treatment and disease status	
Untreated, metastatic	2 (12.5)
Treated and recurred	14 (87.5)
Surgery and adjuvant chemoradiotherapy	5 (31.3)
(chemotherapeutic agent)	
CDDP	3 (18.8)
Cetuximab	1 (6.3)
S-1	1 (6.3)
Surgery and adjuvant radiotherapy	6 (37.5)
Surgery alone	3 (18.8)

Abbreviations: ECOG PS, Eastern Cooperative Oncology Group performance status. SDC, salivary duct carcinoma. IHC, immunohistochemistry. CDDP, cisplatin.

^a^N is the number of patients in the SAS.

^b^Data were presented as the number of patients except for age. The percentage was calculated using N as the denominator.

### Treatment delivery

Of the 16 patients in the SAS, the median dosing period was 175.0 days (25 weeks) for both trastuzumab and docetaxel (Table S1). Fifteen and 14 patients completed eight cycles of trastuzumab and docetaxel, respectively (Table S2). One patient discontinued trastuzumab, and two patients discontinued docetaxel due to AEs. No patients discontinued the treatment due to PD. The treatment schedule was delayed in 11 patients, and dose reduction of docetaxel was reported in eight patients. The median (range) and mean RDI of trastuzumab were 0.96 (range 0.88–1.01) and 0.96, respectively. Those of docetaxel were 0.93 (range 0.71–1.00) and 0.90, respectively. Details regarding treatment discontinuation, delays, and dose reductions due to AEs were described in the Safety section.

Of the 16 patients in the SAS, 12 received subsequent treatment (Table S3). Four patients received maintenance therapy following protocol treatment; two received trastuzumab and docetaxel, and two, docetaxel alone. These treatments were carried out at the strong request of the patients, and new informed consent was verbally obtained. Regarding the cost of treatment after the protocol therapy, docetaxel alone was administered under the national health insurance system. Among the patients who received combination therapy with trastuzumab and docetaxel, one was treated under the public insurance system without reimbursement denial by submitting a detailed medical report, as was accepted in that region of Japan at the time, and the other received treatment on a self-pay basis. Ten patients received treatment after the progression of protocol treatment, nine of whom received systemic therapy with various anti-tumor drugs. No treatment other than maintenance therapy was started before the progression of protocol treatment.

### Efficacy

**Figure 2:**
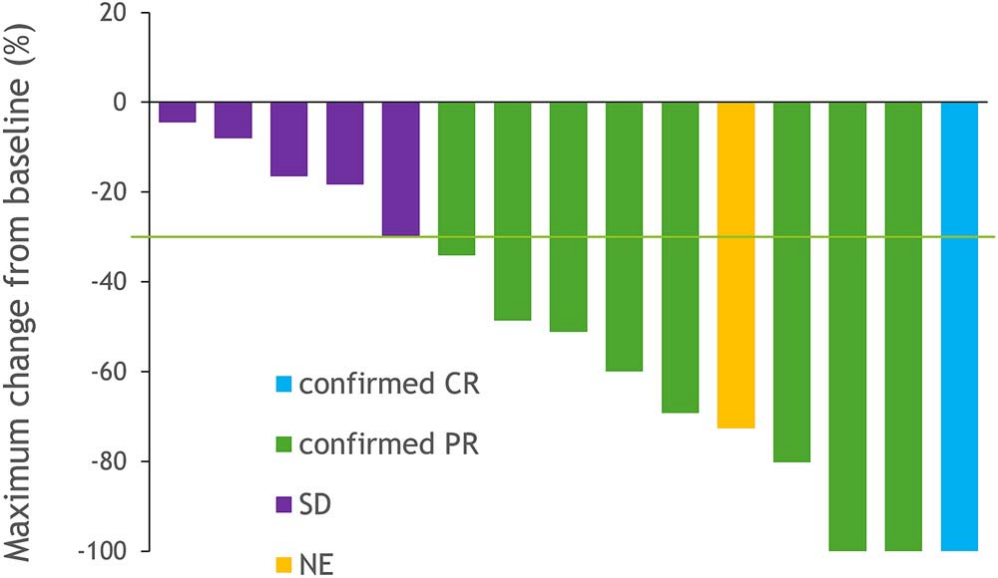
The best reduction from baseline in target lesions. Tumor shrinkage relative to baseline was observed in all patients (100%). The line represents the threshold for a PR. Abbreviations: CR, complete response. PR, partial response. SD, stable disease. PD, progressive disease. NE, not evaluable.

Of the 16 patients who received trastuzumab and docetaxel, one was excluded from the FAS because the patient was assessed to have no measurable lesion by BIRC (Fig. 1). This excluded case had target lesions according to the investigator’s assessment and was determined to have achieved a PR; therefore, the protocol treatment was completed. For the 15 patients in the FAS, the confirmed ORR determined by the BIRC was 60.0% (95% CI, 32.3 to 83.7) (Table 2). One patient (6.7%) achieved a CR, and eight (53.3%) exhibited a PR. Five patients had SD. One patient showed SD at the initial imaging on day 36, but died before the second assessment could be performed, failing to meet the 6-week confirmation period; the best overall response was therefore classified as NE. No patients had PD. The DCR was 93.3% (95% CI, 68.1 to 99.8). Tumor shrinkage was observed in all patients (Fig. 2).

**Table 2:** Response based on the best overall response determined by a BIRC

Response assessment by BIRC	N = 15^a^
Response, n (%)^b^	
Confirmed CR	1 (6.7)
Confirmed PR	8 (53.3)
SD	5 (33.3)
PD	0 (0)
NE	1 (6.7)
Confirmed ORR, % (95% CI)	60.0% (32.3–83.7)
DCR, % (95% CI)	93.3% (68.1–99.8)

Abbreviations: BIRC, blinded independent review committee. CR, complete response. PR, partial response. SD, stable disease. PD, progressive disease. NE, not evaluable. ORR, overall response rate (CR + PR). DCR, disease control rate (CR + PR + SD).CI, confidence interval.

^a^N is the number of patients in the full analysis set.

^b^Data were presented as the number of patients. The percentage was calculated using N as the denominator.

**Figure 3:**
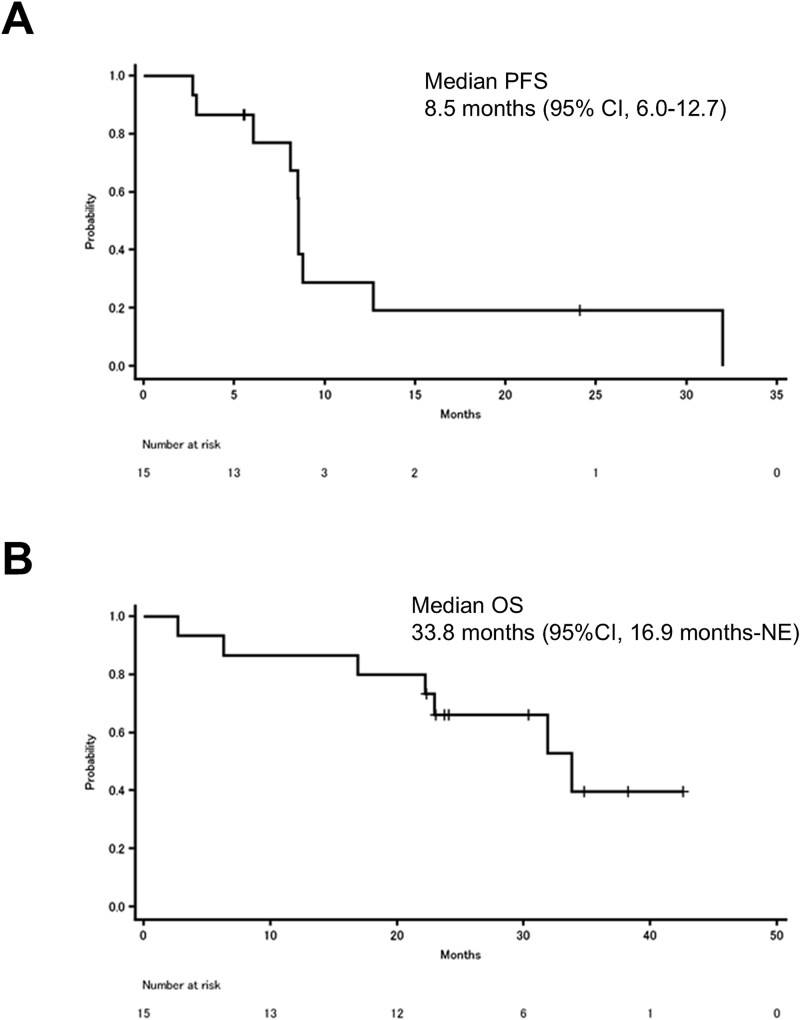
Kaplan–Meier plots of PFS (A) and OS (B). Tick marks indicate censored events. Abbreviations: PFS, progression-free survival. OS, overall survival. NE, not estimable. CI, confidence interval.

The median PFS was 8.5 months (95% CI, 6.0 to 12.7) (Fig. 3A). The median OS was 33.8 months (95% CI, 16.9 to not estimable), with a median follow-up of 23.7 months (Fig. 3B). According to the protocol, the PFS for four patients who received trastuzumab plus docetaxel or docetaxel alone as maintenance therapy was censored before the initiation of the maintenance therapy.

**Figure 4:**
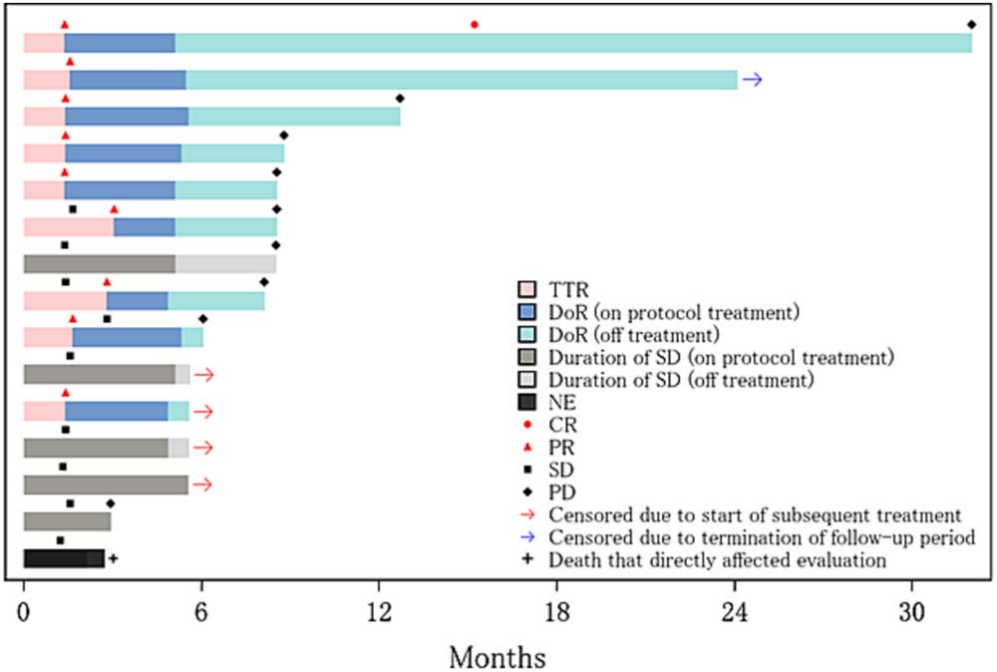
Swimmer plot of treatment duration and response outcomes. Each bar represents an individual patient. Colored segments indicate the TTR, DoR, duration of SD. For patients whose response was not evaluable (NE), the corresponding period is marked as NE. Symbols indicate overall response: CR, PR, SD, and PD. The two types of arrows indicate censoring due to the start of subsequent treatment and the termination of follow-up period, respectively. Plus sign indicates death that directly affected evaluation (i.e. death occurred before PD assessed by RECIST v1.1 and clinical progression).

The median TTR was 1.41 months (95% CI: 1.28–2.28). The median DoR during the on-protocol treatment period was 3.71 months (95% CI: 2.81–4.01), and the median DoR after the termination of protocol treatment was 3.45 months (95% CI: 0.54–14.5). When combining both periods, the median DoR was 7.16 months (95% CI: 3.74–18.11). A swimmer plot illustrating individual patient treatment durations and response outcomes is shown in Fig. 4. As with PFS, DoR and duration of SD were censored at the initiation of the maintenance therapy.

### Safety

TEAEs of any grade and grade ≥3 occurred in all 16 patients (Tables 3, 4, and  S4). The most frequent TEAEs (incidence of 50% or more) were neutrophil count decreased, white blood cell count decreased, alopecia, anemia, hypoalbuminemia, stomatitis, peripheral edema, and nail disorder. The most frequent grade 3 or higher TEAEs (incidence of 10% or more) were neutropenia (100%), leukopenia (93.8%), lymphopenia (18.8%), and febrile neutropenia (12.5%) (Tables 3, 4, S4, and  S5). Serious TEAEs and TRAEs were reported in seven patients (43.8%), including one patient with grade 5 TRAE of hypoalbuminemia (Tables 4, S6, and  S7). The patient had mild hypoalbuminemia due to diabetic nephropathy before the trial, which gradually worsened after the start of the trial and rapidly exacerbated after the fourth cycle. The patient succumbed to respiratory failure due to pleural effusion associated with hypoalbuminemia. Details of the clinical course are provided in Supplementary S6. TEAEs of cardiac disorders were only grade 1 palpitation (6.3%), and neither cardiac failure nor decreases in left LVEF occurred.

**Table 3:** Common treatment-emergent AEs by worst grade

	**N = 16** ^**a**^				
	**Number of patients by worst grade** ^**b**^				
**TEAEs (PT)** ^**c**^	**All grades (%)**	**G3**	**G4**	**G5**	**G3 ≤ (%)**
Any events	16 (100)	2	13	1	16 (100)
Hematologic					
Neutrophil count decreased	16 (100)	2	14	0	16 (100)
White blood cell count decreased	15 (93.8)	10	5	0	15 (93.8)
Anemia	13 (81.3)	1	0	0	1 (6.3)
Lymphocyte count decreased	5 (31.3)	3	0	0	3 (18.8)
Febrile neutropenia	2 (12.5)	2	0	0	2 (12.5)
Non-hematologic					
Alopecia	14 (87.5)	0	0	0	0 (0)
Hypoalbuminemia	10 (62.5)	0	0	1	1 (6.3)
Stomatitis	10 (62.5)	0	0	0	0 (0)
Peripheral edema	8 (50.0)	0	0	0	0 (0)
Nail disorder	8 (50.0)	0	0	0	0 (0)
Anorexia	7 (43.8)	1	0	0	1 (6.3)
Malaise	7 (43.8)	0	0	0	0 (0)
Skin rash	7 (43.8)	0	0	0	0 (0)
Weight gain	6 (37.5)	0	0	0	0 (0)
LDH increased	5 (31.3)	0	0	0	0 (0)
Pleural effusion	5 (31.3)	0	0	0	0 (0)
ALT increased	5 (31.3)	0	0	0	0 (0)
Peripheral sensory neuropathy	4 (25.0)	0	0	0	0 (0)
Fever	4 (25.0)	0	0	0	0 (0)
Hypocalcemia	4 (25.0)	0	0	0	0 (0)
Infusion reaction	4 (25.0)	0	0	0	0 (0)
Cough	4 (25.0)	0	0	0	0 (0)
Diarrhea	4 (25.0)	0	0	0	0 (0)
Bronchitis	2 (12.5)	1	0	0	1 (6.3)
Nausea	2 (12.5)	1	0	0	1 (6.3)
Dysphagia	1 (6.3)	1	0	0	1 (6.3)
Insomnia	1 (6.3)	1	0	0	1 (6.3)
Lung infection	1 (6.3)	1	0	0	1 (6.3)
Urine output decreased	1 (6.3)	1	0	0	1 (6.3)
Hypokalemia	1 (6.3)	1	0	0	1 (6.3)
Anal fistula	1 (6.3)	1	0	0	1 (6.3)
Hyperglycemia	1 (6.3)	1	0	0	1 (6.3)
Aspiration pneumonia	1 (6.3)	1	0	0	1 (6.3)

Abbreviation: TEAEs, treatment-emergent adverse events. PT, preferred term. G, grade.

^a^N is the number of patients in the SAS.

^b^Data were presented as the number of patients. The percentage was calculated using N as the denominator. TEAEs that occurred in at least 20% and those of grades ≥3 are shown.

^c^The AEs were presented by PT using MedDRA/J version 22.0.

**Table 4:** Overall safety summary

	**N = 16** ^**a**^
**Type of AE**	**Number of patients (%)** ^**b**^
TEAEs	16 (100)
Treatment-related	16 (100)
TEAEs grade ≥ 3	16 (100)
Treatment-related	16 (100)
SeriousTEAEs	7 (43.8)
Treatment-related	7 (43.8)
TEAEs leading to death	1 (6.3)
Treatment-related	1 (6.3)
TEAEs leading to treatment discontinuation	
Trastuzumab	1 (6.3)
Treatment-related	1 (6.3)
Docetaxel	2 (12.5)
Treatment-related	2 (12.5)
TEAEs leading to administration delay^**c**^	
Trastuzumab	7 (43.8)
Treatment-related	6 (37.5)
Docetaxel	6 (37.5)
Treatment-related	5 (31.3)
TEAEs leading to infusion interruption	
Trastuzumab	1 (6.3)
Treatment-related	1 (6.3)
Docetaxel	1 (6.3)
Treatment-related	1 (6.3)
TEAEs leading to dose reduction	
Docetaxel	8 (50.0)
Treatment-related	8 (50.0)

Abbreviations: N: number of patients in the SAS. AE, adverse event. TEAEs, treatment-emergent adverse events.

^a^N is the number of patients in the SAS.

^b^Data were presented as the number of patients. The percentage was calculated using N as the denominator.

^c^If the administration of one of the drugs is delayed, the administration of the other drug should also be delayed to ensure they are administered on the same day.

Both trastuzumab and docetaxel were discontinued in one patient due to the grade 5 hypoalbuminemia, and docetaxel alone in one patient due to grade 1 pneumonitis (Table 4, S8, and  S9). TEAEs and TRAE led to administration delays of both drugs in ~30%–40% of patients (Tables S10–S13). TEAEs causing infusion interruptions occurred in 6.3% of patients for both trastuzumab and docetaxel, all treatment-related (Tables S14 and S15). TEAEs also resulted in dose reductions of docetaxel in 50.0% of patients, all treatment-related.

Primary prophylaxis with G-CSF during the first course was not used in any patients, while secondary prophylaxis with G-CSF was administered to seven patients, five of whom received PEG-GCSF. The therapeutic use of G-CSF was applied to 11 patients. Prophylactic antibiotics were also administered in five patients: three received levofloxacin, and one patient each received amoxicillin, garenoxacin, and cefepime. Dose reductions were made in both patients (12.5%) with febrile neutropenia and in two patients (12.5%) with grade 4 neutropenia (Table S16). Treatment delays due to neutropenia or febrile neutropenia occurred in one patient (6.3%) with grade 4 neutropenia (Tables S12 and S13).

## Discussion

In this multicenter phase II trial, we investigated the efficacy and safety of trastuzumab in combination with docetaxel for HER2-positive RM SGC, pursuant to J-GCP. The BIRC-assessed confirmed ORR of 60.0% (95% CI, 32.3 to 83.7%) demonstrated that the lower limit of the 95% CI exceeded the threshold of 25%, surpassing the ORR of chemotherapy observed in phase II studies of SGC [5–12]. The safety results were consistent with the known safety profiles of trastuzumab and docetaxel reported for SGC [24] and breast cancer [25], and the toxicity was considered manageable. This combination therapy showed promising clinical benefits for this patient population, leading to the simultaneous approval of trastuzumab and HER2 companion diagnostics IHC/DISH assay for SGC in Japan, marking a global first.

The findings align with and expand upon previous studies on HER2-targeted therapies in SGC. In a phase II trial of trastuzumab as a single agent in HER2-positive SGC, ORR was only 7% [21], underscoring the limited efficacy of monotherapy in this setting. In contrast, the combination of trastuzumab and docetaxel achieved a significantly higher ORR of 60.0% in this multicenter study, effectively replicating the 70.2% ORR reported in the single-institute phase II trial of the same regimen [24], and the 69.8% ORR in the phase II trial of trastuzumab biosimilar and docetaxel polymeric micelle [26], for HER2-positive SGC. The superiority of trastuzumab and docetaxel to trastuzumab monotherapy is consistent with a phase III trial comparing these treatments for HER2-positive breast cancer, which showed a marked difference in median PFS: 445 days for the trastuzumab and docetaxel group versus 114 days for trastuzumab monotherapy (hazard ratio [HR], 4.24; P < .01) [27]. This consistency across different cancers further supports the enhanced efficacy of the combination therapies.

Other HER2-targeted combination therapies have also demonstrated promising results. T-DM1 [28] and the combination of pertuzumab and trastuzumab [29] achieved ORR of 90.0% and 60.0% in a small subgroup of HER2-positive SGC patients in basket trials, respectively. These findings highlight the enhanced efficacy of antibody-drug conjugates or dual-targeted approaches compared to trastuzumab monotherapy. Additionally, T-DXd has shown an ORR of 58.8% in a pooled analysis of two phase I studies for patients with HER2-expressing SGC (IHC ≥ 1+) [30], and has recently received FDA approval for HER2-positive (limited to IHC 3+) solid tumors based on the results from a basket phase II trial [31]. The high ORRs observed in these trials, including our trial, suggest that HER2-targeted therapies, particularly when combined with other agents or used as antibody-drug conjugates, can significantly improve outcomes for patients with HER2-positive SGC.

In addition to the high ORR, the tumor shrinkage in all patients and the high DCR of 93.3% underscore the significant therapeutic potential of this treatment. The median PFS of 8.5 months and median OS of 33.8 months are also favorable, given the aggressive nature of recurrent metastatic SGC and phase II trials primarily targeting SGC, which show a median PFS of 4 to 6 months for chemotherapy [9,10], and 8.9 months for HER2-targeted therapy [24]. Furthermore, in this study, PFS for four patients who received trastuzumab plus docetaxel or docetaxel alone as maintenance therapy was censored before the initiation of the maintenance therapy, which may have led to an underestimation of the PFS in this trial, as indicated by the swimmer plot.

Although this trial limited the number of protocol treatment cycles to a maximum of eight due to funding constraints in investigator-initiated clinical trials, four patients continued the treatment as post-protocol maintenance therapy. The median and maximum numbers of cycles in the prior trial for SGC, using the same dosage as this trial, were 10.5 and 36, respectively [24]. In a phase III trial for HER2-positive inoperable or recurrent breast cancer, the median and maximum numbers of cycles in the trastuzumab and docetaxel group were 8.0 and 41, respectively [25]. The prolonged efficacy and tolerable toxicity shown in those trials suggest no need to limit the treatment to eight cycles.

The majority of grade ≥ 3 TEAEs were hematologic, primarily neutropenia, which aligns with the known toxicity profile of trastuzumab and docetaxel [24,25]. TEAEs of any grade included mainly hematologic and gastrointestinal toxicities, along with alopecia, hypoalbuminemia, stomatitis, peripheral edema, and nail disorder, most of which were grade 1 or 2 and manageable. The overall safety profile was consistent with previous reports, with no indication of increased severe or fatal AEs [24,25].

Nonetheless, serious TEAEs and TRAEs were observed in 43.8% of patients, including two with febrile neutropenia (12.5%) and 1 with grade 5 hypoalbuminemia (6.3%). These findings underscore the importance of careful management, including dose reductions and treatment delays, to maintain tolerability. The patient who died had pre-existing hypoalbuminemia due to diabetic nephropathy, which worsened during the trial and rapidly deteriorated after the fourth cycle, possibly related to the study drug. Although hypoalbuminemia alone is an uncommon direct cause of mortality, this AE was considered to have contributed to the accumulation of bilateral pleural effusion, leading to worsening respiratory status and death, based on no findings suggestive of carcinomatous or infectious pleuritis, heart failure, or drug-induced pneumonitis. Considering its characteristics, the pleural effusion was managed with albumin supplementation and diuretic agents. This case highlights the importance of careful consideration of hypoalbuminemia when administering this treatment, particularly in patients with comorbidities or baseline hypoalbuminemia.

Given the relatively low incidence of febrile neutropenia at 12.5%, primary prophylaxis with G-CSF is currently considered unnecessary [32], while appropriate dose reductions and treatment delays are essential. Our results also suggest that prophylactic antibiotic administration and secondary prophylaxis with G-CSF may contribute to safety and could be further assessed.

Despite the encouraging results, several limitations must be acknowledged. The small sample size and the relatively short follow-up period for some patients limit the generalizability of the findings. Potential bias may include SGC subtypes and HER2 status. Larger trials are needed to confirm these results and better understand this combination therapy’s long-term efficacy and safety. Furthermore, the ~10-year interval between the start of the study and the publication of its results has limited the novelty of this research.

In conclusion, the multicenter phase II trial of trastuzumab and docetaxel demonstrates significant clinical benefits in patients with HER2-positive RM SGC. The high ORR, prolonged PFS, and manageable safety profile highlight the potential of this combination therapy as a new standard of care, leading to the simultaneous approval of trastuzumab and HER2 companion diagnostics assay for this setting in Japan. These findings underscore the importance of continued research and clinical trials to improve outcomes for patients with this rare and challenging disease. We are currently investigating the efficacy of T-DXd for refractory SGC with HER2-positive (IHC 3+ or IHC 2+/ISH +) SGC as well as HER2-low (IHC 2+/ISH—or IHC 1+) SGC in a multicenter phase II clinical trial (jRCT2011210017).

## Supplementary Material

Supplementary_materials_hyaf106

Supplementary_tables_hyaf106
