# Supplementary material for: Multicenter phase II trial of trastuzumab and docetaxel for HER2-positive salivary gland cancer
Source: Jpn J Clin Oncol. 2025 Jun 25;55(10):1131–40. doi: 10.1093/jjco/hyaf106 (PMC12501972; doi:10.1093/jjco/hyaf106)
Supplement: Supplementary_materials_hyaf106 [file supplementary_materials_hyaf106.docx]

Supplementary documents

[Doc. S1. Full eligibility criteria 2](#_Toc15974)

[Doc. S2. Protocol treatment 6](#_Toc15975)

[Doc. S3. Criteria for treatment modification 8](#_Toc15976)

[Doc. S4. Criteria for termination or discontinuation of protocol treatment 16](#_Toc15977)

[Doc. S5. Regulations on concomitant drugs and therapies 19](#_Toc15978)

[Doc. S6. Final clinical study report narrative - fatal case 21](#_Toc15978)

1

# Doc. S1. Full eligibility criteria

## Full eligibility criteria

The patients eligible to receive the investigational drug in this clinical trial are those who are currently attending or admitted to the medical institutions listed in “the List of Medical Institutions Conducting the Clinical Trial," meet the inclusion criteria, and are not subject to any of the exclusion criteria in the following sections.

## 1 Inclusion criteria

1. Patients aged 20 to 75 years when obtaining consent.
2. Patients histologically diagnosed with salivary gland cancer at the central assessment laboratory (Department of Surgical Pathology, Hokkaido University Hospital).
3. Patients with recurrent or metastatic salivary gland cancer.
4. Patients deemed incurable by radical treatment by the principal investigator or sub-investigator.
5. Patients for whom tumor tissue blocks or unstained slides are available for submission to the central assessment laboratory for HER2 overexpression and gene amplification testing.
6. Patients with tumor samples evaluated by the central assessment laboratory who demonstrated HER2 overexpression with an immunohistochemistry (IHC) score of 3+ (IHC-3+) or IHC 2+ with HER2 gene amplification (HER2/CEP17 ratio of 2.0 or higher) detected by dual in situ hybridization (DISH).
7. Patients with measurable lesions according to RECIST v1.1 (Japanese version JCOG v1.0).
8. Patients with an ECOG Performance Status (PS) of 0 to 2.
9. Patients expected to survive for at least three months during the trial period.
10. Patients who have received a sufficient explanation about participation in the trial, understood it well and provided written informed consent of their free will.
11. Patients with the latest test values within 14 days before registration (the same weekday two weeks before the registration date is acceptable) meeting all of the following:
    1. Neutrophil count ≥ 1500/mm³
    2. Hemoglobin ≥ 8.0 g/dL
    3. Platelet count ≥ 10×10⁴/mm³
    4. Total bilirubin ≤ 1.5 mg/dL
    5. AST ≤ 2.5 × upper limit of normal (ULN)
    6. ALT ≤ 2.5 × upper limit of normal (ULN)
    7. Serum creatinine ≤ 2.0 × upper limit of normal (ULN)
    8. SpO2 ≥ 90% (room air)

[Rationale for inclusion criteria]

1. To select patients who can appropriately consent to participate in the trial and whose efficacy and safety can be adequately evaluated.
2. To confirm efficacy in patients diagnosed with salivary gland cancer.
3. To confirm efficacy in patients with recurrent or metastatic salivary gland cancer.
4. To confirm efficacy in patients with poor prognosis for whom standard treatments, such as surgical resection and radiotherapy for salivary gland carcinoma, are not feasible.

5.-6. To confirm efficacy in patients with HER2-positive salivary gland cancer.

1. To appropriately evaluate the investigational drug.
2. To ensure the safety of subjects by excluding those with a deteriorated general condition, often accompanied by various symptoms.
3. To select patients whose efficacy and safety can be adequately evaluated.
4. To ensure ethical participation based on the subject's free will.
5. To confirm that patients maintain adequate organ function when implementing the trial.

## 2 Exclusion criteria

1. Patients with prior experience using taxane-based anticancer drugs (docetaxel, paclitaxel). However, patients were not excluded if they had completed curativeintent chemotherapy (including induction chemotherapy, chemoradiotherapy, or postoperative adjuvant chemotherapy) more than six months before enrollment.
2. Patients who have previously used trastuzumab.
3. Patients who received other anticancer therapies within four weeks before registration. However, treatment with bisphosphonates or RANK ligand inhibitors for pre-existing bone metastases or osteoporosis was allowed (preventive use in patients without bone metastases was prohibited).
4. Patients meeting any of the following criteria:
   - History of congestive heart failure.
   - Angina requiring medication.
   - History of myocardial infarction.
   - Uncontrolled hypertension (systolic blood pressure ≥ 160 mmHg or diastolic blood pressure ≥ 100 mmHg).
   - Clinically significant valvular heart disease.
   - High-risk uncontrolled arrhythmias.
5. Patients with a left ventricular ejection fraction (LVEF) < 50% on echocardiography or multi-gated acquisition (MUGA) scan within 28 days before registration.
6. Patients with cardiovascular disease classified as NYHA II, III, or IV.
7. Patients treated with anthracyclines exceeding a cumulative dose of 500 mg/m² of doxorubicin.
8. Patients diagnosed with interstitial pneumonia or pulmonary fibrosis by chest Xray or CT scan or with a history of these conditions.
9. Patients with advanced malignancies or other diseases causing dyspnea at rest or requiring oxygen therapy.
10. Patients with a history of drug allergies deemed problematic for participation in the trial.
11. Patients with active infections deemed problematic for participation in the trial.
12. Patients with Grade 2 or higher motor paralysis or peripheral neuropathy.
13. Patients with Grade 2 or higher edema.
14. Patients requiring systemic corticosteroids (prednisone equivalent > 10 mg/day) or other immunosuppressive therapy within 14 days before the initial administration (inhaled steroids and adrenal cortex steroid replacement therapy [prednisone equivalent > 10 mg/day] were allowed if there was no active autoimmune disease).
15. Patients with other severe comorbidities (e.g., cerebrovascular disorders, active peptic ulcer, uncontrolled diabetes mellitus, clinically significant psychiatric or neurological disorders).
16. Patients positive for hepatitis B surface antigen (HBsAg).
17. Patients with effusions (pleural effusion, ascites, pericardial effusion) requiring drainage (eligible if at least two weeks have passed since drainage and pleurodesis).
18. Patients who received radiation therapy within four weeks before registration (eligible if palliative radiation therapy was performed on peripheral bone metastases, the patient recovered from all acute toxicity, and at least two weeks passed).
19. Patients who underwent major surgery within four weeks before registration (eligible if fully recovered).
20. Patients with active concurrent malignancies (including synchronous or metachronous cancers with a disease-free interval of less than three years, excluding carcinoma in situ or equivalent mucosal lesions considered cured by local treatment).
21. Pregnant or lactating women, women with the possibility or intent of pregnancy, and patients wishing their partner to become pregnant.
22. Patients with severe alcohol intolerance or allergy.
23. Other patients deemed unsuitable for participation in the trial by the principal investigator or sub-investigator.

[Rationale for exclusion criteria]

1.-3. 20. To exclude factors affecting the evaluation of the investigational drug.

4.-19. 21.-23. To minimize risks and ensure the safety of subjects.

# Doc. S2. Protocol treatment

## Protocol treatment

Trastuzumab administration should commence within two weeks of patient enrollment in the protocol treatment. Trastuzumab, combined with docetaxel, will be administered intravenously according to the following regimen, with each cycle lasting three weeks (21 days). On day 1 of each cycle, trastuzumab will be administered first, followed by docetaxel. In the first cycle, trastuzumab (8 mg/kg) will be administered intravenously over 90 minutes, followed by an observation period of at least 1 hour after

administration. If no infusion reaction occurs during the first cycle, observation will not be necessary for subsequent cycles. However, if an infusion reaction occurs from the second cycle onward, a 1-hour observation period will be reinstated. After the observation period, docetaxel (70 mg/m²) will be administered intravenously over approximately 60 minutes. As a rule, trastuzumab and docetaxel should be administered on the same day. However, if an infusion reaction makes same-day administration difficult, administration on the following day is permissible.

The protocol treatment will be conducted for a maximum of 8 cycles. If the administration of either trastuzumab or docetaxel is discontinued before completing three cycles, the protocol treatment will be terminated. If the administration of trastuzumab and docetaxel continues for three or more cycles, it is permissible to continue with either trastuzumab or docetaxel alone from the fourth cycle onward when one of the drugs is discontinued. Additionally, a delay in administration of up to 42 days from the last administration of either trastuzumab or docetaxel is permissible. If one of the drugs is delayed, the other drug's administration should also be delayed, ensuring they are administered on the same day.

It is acknowledged that precise 3-week intervals for administration may not always be feasible due to holidays, outpatient visits, or other circumstances. In such cases, a delay of up to 7 days from the scheduled start date of the next cycle is permissible. Delays of 8 days or more from the scheduled start date of the next cycle should be avoided as much as possible.

Furthermore, as this is an investigator-initiated trial, providing trastuzumab under the compassionate use system (which allows the use of unapproved drugs) is challenging due to funding constraints. Therefore, the protocol treatment will conclude after a maximum of 8 cycles.

# Doc. S3. Criteria for treatment modification

## Criteria for treatment modification

The following terms will be used for the modification criteria (Table S3)

Table S3

| Delay | Extending the interval between doses due to not meeting the criteria for starting the next dose or for reasons related to the patient's convenience. |
| --- | --- |
| Infusion interruption | Temporarily stopping the administration due to abnormalities such as an infusion reaction. |
| Discontinuation | Permanently stopping the entire treatment or a specific drug, with no plans for resumption. |

## 1 Administration of trastuzumab

1.1 Method of administration

Trastuzumab is administered intravenously once daily. For the initial dose, 8 mg/kg (body weight) is administered over 90 minutes or more. For subsequent doses, 6 mg/kg is administered. If the initial dose is welltolerated, the administration time for subsequent doses may be reduced to 30 minutes. The body weight recorded during the screening test will be used to calculate the initial dose. The dose will be recalculated only if a weight change is 10% or more from the screening weight.

1.2 Criteria for initiating the trasutuzumab (see Table S3.1.2)

1. At the time of trasutuzumab administration, on each scheduled administration day or the day before, it must be confirmed that the riteria for initiating the administration outlined in the table below are met.
2. If the criteria are not met, the administration of the next cycle should be delayed until the criteria are met.
3. Additionally, if deemed necessary by the attending physician, the administration of the next cycle may be delayed.
4. If the criteria are not met within 42 days (21 days from the scheduled start of the next cycle), the administration of trastuzumab for that particular case should be discontinued.

Table S3.1.2

| **Item** | **Criteria for Iiitiating trasutuzuma administration** |
| --- | --- |
| PS | 0 – 2 |
| Heart Failure | No apparent symptoms or findings (NYHA Class II or lower) |
| LVEF | ≥ 50% if an echocardiogram is performed (provided the decrease is less than 20 percentage points from the initial administration) |

■NYHA (New York Heart Association) classification of heart failure

| I | Ordinary physical activity does not cause undue fatigue, dyspnea, or palpitations. |
| --- | --- |
| II | Ordinary physical activity causes fatigue, dyspnea, palpitations, or angina. |
| III | Comfortable at rest; less than ordinary physical activity causes fatigue, dyspnea, palpitations, or angina. |
| IV | Symptoms occur at rest; any physical activity increases discomfort. |

- 1. Criteria for modifying the dosage of trastuzumab

Even if the administration of trastuzumab is delayed, the dosage will not be reduced upon resumption.

・ If the administration is delayed up to one week from the scheduled date, administer 6 mg/kg.

・ If the administration is delayed by more than one week from the scheduled date, administer the initial dosage of 8 mg/kg again, and for subsequent administrations, administer 6 mg/kg at three-week intervals.

・

- 1. Infusion reaction

The definition of an infusion reaction related to trastuzumab follows CTCAE v4.03. Symptoms occurring during or after trastuzumab administration are defined according to adverse event definitions, such as allergic reactions, anaphylaxis, or cytokine release syndrome (CTCAE v4.03 “Immune System Disorders”).

Infusion reactions (symptoms: fever, chills, nausea, vomiting, pain, headache, cough, dizziness, rash, asthenia, etc.) that occur frequently during or within 24 hours after trastuzumab administration are usually mild to moderate and most commonly appear during the first administration of the drug.

Patients should be closely monitored. If any abnormalities are observed, trastuzumab administration should be interrupted, appropriate measures (e.g., antipyretic analgesics, antihistamines) should be taken, and the patient should be closely monitored until symptoms resolve.

After symptoms disappear, re-administration at a slower infusion rate may be possible, but this should be determined based on the patient's condition. Symptoms are less likely to occur after the second administration.

Although the usefulness of premedication with antipyretic analgesics, antihistamines, or steroids has not been confirmed, it can be administered at the discretion of the attending physician.

If severe adverse effects such as anaphylaxis-like symptoms or pulmonary disorders (e.g., bronchospasm, severe hypotension, acute respiratory distress syndrome) occur among the infusion reactions, trastuzumab administration should be immediately discontinued, appropriate measures (e.g., oxygen inhalation, β-agonists, corticosteroids) should be taken, and the patient should be closely monitored until symptoms resolve. Re-administration should not be attempted.

## 2 Administration of docetaxel

2.1 Method of administration of docetaxel

The principal investigator or sub-investigator should thoroughly refer to the package insert for docetaxel and follow the procedures of the medical institution where the study is being conducted. Docetaxel should be administered intravenously once daily at 70 mg/m² (body surface area) over approximately 60 minutes. Docetaxel administration must not begin until the 1-hour observation period following the completion of trastuzumab administration in the first cycle has ended. If no infusion-related reactions are observed during the first cycle of trastuzumab administration, subsequent cycles do not require an observation period, and docetaxel can be administered immediately after trastuzumab. If an infusion reaction occurs from the second cycle onwards, a 1-hour observation period will be reinstated in the next cycle.

The dosage will be recalculated only if there is a weight change of 10% or more from the screening weight. Docetaxel should be administered according to standard methods (e.g., the package insert or the procedures of the medical institution).

2.2 Criteria for initiating docetaxel (see Table S3.2.2.)

1. At the time of docetaxel administration, on each scheduled administration day or the day before, it must be confirmed that the criteria for initiating the administration outlined in the table below are met.
2. If the criteria are not met, the administration of the next cycle should be delayed until the criteria are met.
3. Additionally, if deemed necessary by the attending physician, the administration of the next cycle may be delayed.
4. If the criteria are not met within 42 days (21 days from the scheduled start of the next cycle) from the last docetaxel administration, the administration of docetaxel for that particular case should be discontinued.

Table S3.2.2

| **Item** | **Criteria for initiating docetaxel administration** |
| --- | --- |
| PS | 0-2 |
| Neutrophil count | ≥ 1,500/mm³ |
| Platelet count | ≥ 100,000/mm³ |
| Total bilirubin | ≤ 1.5 mg/dL |
| AST, ALT | ≤ 2.5 × Upper Limit of Normal (ULN) of the institution's standard values |
| ALP | ≤ 2.5 × Upper Limit of Normal (ULN) of the institution's standard values |
| Cr | ≤ 2.0 × Upper Limit of Normal (ULN) of the institution's standard values |
| Other non- | Grade 0 – Grade 2 (Excluding constipation, anorexia, |
| **Item** | **Criteria for initiating docetaxel administration** |
| hematologic toxicities | nausea, alopecia, pigmentation, taste disorder, generalized fatigue, abnormal clinical laboratory values, and other adverse events that the attending physician determines do not require delaying or reducing the dose) |

2.3 Criteria for modifying the dosage of docetaxel (see Table S3.2.3)

If the dose is reduced, all subsequent cycles should be administered at the reduced dose unless further dose reduction is required. Dose reescalation is not allowed.

Dose modification should be based on the worst hematologic test values and the most severe non-hematologic toxicities observed in the previous cycle.

Docetaxel administration starts at 70 mg/m². If the following toxicities occur, administration should be delayed until the criteria for initiating administration (see Table S3.2.3) are met and then resumed at a reduced dose of 55 mg/m².

Table S3.2.3

| **Criteria for toxicities requiring dose reduction of docetaxel** |
| --- |
| Febrile neutropenia during the administration period (ANC < 1.0×10³/μL and either a single temperature > 38.3°C or a sustained temperature > 38°C for more than 1 hour) |
| Neutrophil count < 500/mm³ lasting for more than seven days |
| Total bilirubin > 1.5 mg/dL, and either AST, ALT > 2.5 × Upper Limit of Normal (ULN) of the institution's standard values, or ALP > 2.5  × ULN |
| Platelet count < 25,000/mm³ |
| Severe or persistent skin reactions |
| Non-hematologic toxicities of Grade 3 or higher, other than allergic reactions/anaphylaxis (excluding constipation, anorexia, nausea, alopecia, pigmentation, taste disorders, fatigue, abnormal clinical laboratory values, and other adverse events that the attending physician determines do not require delaying or reducing the dose) |
| **Criteria for toxicities requiring dose reduction of docetaxel** |
| Other cases where dose reduction is deemed necessary by the attending physician |

If any of the above toxicities recur after reducing the dose to 55 mg/m², the administration of docetaxel should be delayed until the criteria for initiating administration (see Table S3.2.2) are met, and then resumed at a further reduced dose of 45 mg/m².

If any of the above toxicities recur after reducing the dose to 45 mg/m², docetaxel should be discontinued without further dose reduction.

If the administration of the next cycle cannot be done within 42 days from the last administration date of the previous cycle (within 21 days from the scheduled date of the next cycle), the administration should be discontinued. Even if docetaxel administration is discontinued, the study can continue with the administration of trastuzumab.

If Grade 3 or 4 nausea or vomiting occurs, antiemetic therapy should be administered, and the dose of docetaxel should remain unchanged, continuing the administration. If nausea and vomiting are not improved by antiemetic treatment, the dose of docetaxel should be reduced.

2.4 Hypersensitivity reactions

Hypersensitivity reactions should be carefully monitored (particularly during the first and second administrations, ensuring thorough observation). Hypersensitivity symptoms can occur within minutes of starting docetaxel administration, so the patient's condition should be closely monitored for one hour after starting the administration. Severe hypersensitivity reactions characterized by generalized rash/erythema, hypotension, bronchospasm, or rare fatal anaphylaxis have been reported even with 3-day premedication with corticosteroids. If a severe hypersensitivity reaction occurs, docetaxel administration should be immediately discontinued, and aggressive treatment should be initiated. Docetaxel should not be re-administered.

Hypersensitivity reactions can occur within minutes of starting docetaxel administration. Mild reactions such as flushing or localized skin reactions do not require interruption of the administration.

**Mild:**

Continue docetaxel administration and monitor the patient. No treatment is necessary.

**Moderate:**

Interrupt docetaxel administration. Administer antihistamines and steroids intravenously. If symptoms resolve, resume docetaxel administration. If symptoms recur, discontinue docetaxel and do not re-administer.

**Severe or life-threatening:**

Discontinue docetaxel administration. Administer antihistamines and steroids intravenously, as with moderate reactions. Additionally, administer epinephrine or bronchodilators if necessary. Do not resume docetaxel administration.

**Administration in subsequent cycles after symptom onset:** If docetaxel is to be administered in subsequent cycles, premedication with antihistamines and steroids 30 minutes before docetaxel administration is recommended, in addition to oral dexamethasone.

2.5 Hematologic toxicity

Neutropenia (neutrophil count < 2000/mm³) occurs in almost all subjects receiving docetaxel at doses of 60–100 mg/m², and Grade 4 neutropenia (neutrophil count < 500/mm³) occurs in 85% of subjects receiving 100 mg/m² and 75% of subjects receiving 60 mg/m². Therefore, it is necessary to frequently monitor blood counts to enable rapid dose adjustment. Docetaxel should not be administered if the neutrophil count is less than 1500/mm³.

2.6 Fluid retention

Severe fluid retention has been reported following docetaxel administration. It is characterized by unacceptable peripheral edema, generalized edema, pleural effusion requiring urgent drainage, dyspnea at rest, cardiac tamponade, and significant abdominal distension (due to ascites). To reduce the incidence and severity of fluid retention, premedication with corticosteroids should be administered at the discretion of the principal investigator or sub-investigator before docetaxel administration. When fluid retention occurs, it usually begins with peripheral edema in the lower extremities and spreads throughout the body, accompanied by weight gain (with a median increase of 2 kg). If pleural effusion is present, careful monitoring for worsening pleural effusion is required from the initial administration of docetaxel.

2.7 Skin

Localized erythema followed by desquamation has been observed, often accompanied by edema of the extremities. If severe skin toxicity occurs, it is recommended that the dose be adjusted.

# Doc. S4. Criteria for termination or discontinuation of protocol treatment

## 1 Criteria for termination of protocol treatment

Protocol treatment shall be considered terminated when eight courses have been completed (refer to section 7.1, "Protocol Treatment").

## 2 Criteria for discontinuation of protocol treatment

Protocol treatment shall be discontinued if any of the following conditions are met:

1. PD (progressive disease) is confirmed based on imaging evaluation by the principal investigator or sub-investigator.
2. PD is confirmed based on worsening the patient's condition.
3. The subject requests discontinuation.
4. It is determined that necessary observations, investigations, or the continuation of the clinical trial are consistently impossible due to the subject's circumstances.
5. The subject is found to be pregnant.
6. After the start of the trial, it is determined that the subject does not meet the inclusion criteria or meet the exclusion criteria, making them unsuitable for the trial.
7. The principal investigator or sub-investigator determines that unacceptable adverse events or toxicity (such as intolerable, persistent moderate toxicity) have occurred, and it cannot be ascertained whether trastuzumab or docetaxel causes these.
8. Regardless of CTCAE v4.03 Grade, a life-threatening event considered related to trastuzumab and docetaxel occurs, and the principal investigator or subinvestigator cannot ascertain whether any other specific drugs caused it.
9. If trastuzumab or docetaxel is discontinued before completing three courses.
10. Any other reason deemed by the principal investigator or sub-investigator to make the continuation of the trial impossible.

### 2.1 Criteria for discontinuation of trastuzumab

The principal investigator and sub-investigator must discontinue administering trastuzumab to the subject if any of the following conditions are met. If the subject has completed three or more courses, they may continue the trial and continue with docetaxel administration.

1. If the administration must be delayed beyond the 42nd day after the last dose of trastuzumab (beyond the 21st day after the scheduled start of the following course).
2. If unacceptable adverse events or toxicity (such as intolerable, persistent moderate toxicity) occur, and the principal investigator or sub-investigator determines that these are caused by trastuzumab.
3. If Grade 3 or 4 hypersensitivity reactions related to injection occur, and the principal investigator or sub-investigator determines that these are caused by trastuzumab.
4. If the left ventricular ejection fraction (LVEF) decreases to less than 40%.
5. If NYHA Class IV or higher heart failure symptoms are observed.

### 2.2 Criteria for discontinuation of docetaxel

The principal investigator and sub-investigator must discontinue the administration of docetaxel to the subject if any of the following conditions are met. If the subject has completed three or more courses, they may continue the trial and continue with the administration of trastuzumab.

1. If a reduction in the dosage of docetaxel is required more than twice.
2. If the administration must be delayed beyond the 42nd day after the last dose of docetaxel (beyond the 21st day after the scheduled start of the following course).
3. If unacceptable adverse events or toxicity (such as intolerable, persistent moderate toxicity) occur, and the principal investigator or sub-investigator clearly determines that these are caused by docetaxel.
4. Regardless of CTCAE v4.03 Grade, if a life-threatening event related to docetaxel occurs.
5. If interstitial pneumonia caused by docetaxel occurs.

## 3 Procedures after discontinuation of protocol treatment

The principal investigator and sub-investigator must take appropriate measures for subjects in whom protocol treatment is discontinued due to safety concerns, such as adverse events. Additionally, all subjects who discontinue treatment must undergo a discontinuation examination within 14 days.

Furthermore, if a subject ceases to visit the trial site after enrollment, follow-up should be conducted to the greatest possible extent, considering the subject's rights and the prescribed evaluations that should be carried out.

The date of discontinuation of protocol treatment shall be when the principal investigator or sub-investigator determines the discontinuation. The principal investigator and sub-investigator must investigate and record the date and reason for the discontinuation in the CRF.

4 **Subsequent therapy after protocol treatment** Subsequent therapy was not predefined.

# Doc. S5. Regulations on concomitant drugs and therapies

## Regulations on concomitant drugs and therapies

From the date of patient registration until four weeks after the completion or discontinuation of protocol treatment, the drugs and therapies listed in 7.3.1. are prohibited. However, the drugs and therapies listed in 7.3.2. are allowed as long as there are no concerns regarding interactions with trastuzumab and docetaxel. If any drugs other than trastuzumab and docetaxel are used from the start of trastuzumab administration until four weeks after the completion or discontinuation of protocol treatment, the drug name, route of administration, dosage, and duration of administration must be recorded in the case report form (CRF).

## 1 Prohibited concomitant drugs and therapies

1. Any anti-tumor therapy (chemotherapy, hormone therapy, immunotherapy, palliative radiotherapy (except for palliative radiotherapy to non-evaluable lesions such as the brain and bones))
2. Potent cytochrome P450 3A4 (CYP3A4) inhibitors
3. Systemic corticosteroids exceeding a prednisone equivalent of 10 mg/day 4) Other investigational drugs [Rationale for the prohibition]

1), 4) Prohibited due to potential impacts on the efficacy and safety evaluation of this clinical trial.

1. Prohibited due to the possibility of increased blood concentration of docetaxel, which could lead to enhanced side effects.
2. Prohibited to ensure patient safety.

## 2 Permitted concomitant drugs and therapies

1. Medications for the treatment or prevention of adverse events
2. Medications for the treatment of comorbidities
3. Medications aimed at symptom relief or blood transfusion therapy
4. Bisphosphonate preparations (use is permitted if bone metastases are present)
5. Denosumab (use is permitted if bone metastases are present)
6. Granulocyte-colony stimulating factor (G-CSF) preparations (in principle, G-

CSF preparations should be administered according to insurance coverage)

1. Local, ocular, intra-articular, intranasal, and inhaled corticosteroids
2. Systemic corticosteroids used at physiological replacement doses (prednisone 10 mg/day or less)
3. Short-term use of corticosteroids for prevention (e.g., contrast agent allergies) or for conditions other than autoimmune diseases (e.g., delayed-type hypersensitivity reactions due to contact allergens)

Doc. S6. Final clinical study report narrative - fatal case

**Case**: 64 years, male

**Comorbidities**: diabetic nephropathy, hypoalbuminemia and anemia

**Clinical course leading to death**:

Day X: The patient received the fourth course of trastuzumab and docetaxel. Non-serious adverse events, including peripheral edema and nausea, persisted. Vital signs were as follows: body temperature 36.5°C, blood pressure 125/68 mmHg, pulse 82 beats/min, respiratory rate 18 breaths/min, and oxygen saturation (SpO_2_) 97% on room air. Laboratory test results showed a white blood cell count of 10.0×10³/μL, hemoglobin 8.7 g/dL, C-reactive protein 0.18 mg/dL, serum creatinine 0.95 mg/dL, albumin 2.0 g/dL, blood urea nitrogen 14.5 mg/dL, estimated glomerular filtration rate 61.9 mL/min/1.73 m², qualitative urine protein 3+, urine glucose ±, and urine occult blood 1+.

Day X+4: Due to generalized fatigue, loss of appetite, reduced oral intake leading to hypoglycemia, and worsening of prerenal renal dysfunction caused by dehydration, the patient was urgently hospitalized. Although chest pain and dyspnea were not present, a decrease of SpO_2_ was observed. Consultations were made with the departments of diabetology and respiratory medicine. Chest CT revealed bilateral pleural effusion, and an infectious cause was considered highly. At that time, interstitial pneumonia was ruled out. Meropenem infusion was initiated.

Day X+5: Although the patient did not experience dyspnea, oxygen was administered at 5 L/min. The hemoglobin level was 6.9 g/dL, and the patient was diagnosed with Grade 3 anemia, for which 2 units of red blood cell concentrate were transfused.

Day X+6: The patient exhibited mild dyspnea and received oxygen therapy at 4–5 L/min (SpO2 93–97%). A thoracentesis was performed, and 60 mL of pleural fluid was drained. The cause of the pleural effusion was under investigation, and consultations with cardiology and nephrology were conducted. Due to a low serum iron level of 13 μg/dL, intravenous administration of ferric derisomaltose was initiated.

Day X+7: No bacteria were detected in the pleural fluid analysis, and bacterial pleuritis was ruled out. The patient’s KL-6 level was 498 U/mL. Echocardiography revealed a small amount of pericardial effusion, and the ejection fraction (EF) was 73.2%. The brain natriuretic peptide level was mildly elevated at 55.7 pg/mL, suggesting that heart failure was unlikely to be the cause of the pleural effusion. The effusion was suspected to result from leakage into the pleural and alveolar spaces due to hypoalbuminemia or increased vascular permeability. According to the nephrology consultation, the proteinuria was attributed to diabetic nephropathy; however, such a rapid onset of hypoproteinemia was not typical in this condition. The hypoalbuminemia was instead considered to result from poor oral intake, loss associated with acute anemia, and decreased hepatic synthetic function. Enteral nutrition was initiated to ensure adequate nutritional support.

Day X+8: The patient's performance status was 4. To treat grade 3 hypoalbuminemia, intravenous administration of 25% albumin at a dose of 12.5 g was initiated.

Day X+9: No malignant cells were detected in the pleural fluid examination. Although a reduction in pleural effusion was observed on chest X-ray, ground-glass opacities were noted. Furosemide was initiated in response to decreased urine output and localized edema.

Day X+10: The patient presented with melena, prompting an upper gastrointestinal endoscopy by the gastroenterology department. The examination revealed non-active upper gastrointestinal bleeding. Enteral nutrition was temporarily discontinued.

Day X+11: The patient’s serum albumin level decreased again to 1.6 g/dL. Hemoglobin dropped to 7.7 g/dL, indicating progression of anemia; therefore, two units of red blood cell concentrate were transfused. Due to sleep disturbances during the night, a consultation with psychiatry was requested.

Day X+15: Enteral nutrition was resumed.

Day X+16: The patient's vital signs remained stable with oxygen administration at 1–2 L/min, and the nighttime sleep disturbance had improved.

Day X+18: The patient experienced a rapid decrease in SpO_2_ and died from respiratory failure due to pleural effusion associated with hypoalbuminemia.

**Principal Investigator’s Assessment**:

Fatigue: Fatigue appeared from the first course of the protocol treatment and gradually worsened with continued treatment. After the initiation of the fourth course, fatigue worsened rapidly, leading to hospitalization. Based on this clinical course, a causal relationship with the protocol treatment could not be ruled out.

Hypoalbuminemia: Mild hypoalbuminemia, presumed to be due to diabetic nephropathy, was present before the start of the clinical trial and gradually worsened after the trial began. Following the initiation of the fourth course, it deteriorated rapidly, and a causal relationship with the protocol treatment could not be excluded. The deterioration of respiratory status due to pleural effusion was considered to be caused by this event.

Anemia: Mild anemia was present before the start of the clinical trial and gradually worsened after initiation. After the fourth course began, it worsened rapidly, and a causal relationship with the protocol treatment could not be ruled out.
