# Supplementary material for: Multicenter phase II trial of trastuzumab and docetaxel for HER2-positive salivary gland cancer
Source: Jpn J Clin Oncol. 2025 Jun 25;55(10):1131–40. doi: 10.1093/jjco/hyaf106 (PMC12501972; doi:10.1093/jjco/hyaf106)
Supplement: Supplementary_tables_hyaf106 [file supplementary_tables_hyaf106.pdf]

Supplementary tables

Table S1. Summary of administration period and cumulative dose of study drugs (SAS) ................ 2
Table S2. Summary of the number of doses, dose delays, interruptions, and reductions (SAS) ...... 3
Table S3. Subsequent treatment (SAS) ........................................................................................ 4
Table S4. All TEAEs by worst grade ........................................................................................... 5
Table S5. All TRAEs by worst grade .......................................................................................... 11
Table S6. Treatment-emergent SAEs by worst grade .................................................................. 15
Table S7. Treatment-related SAEs by worst grade...................................................................... 16
Table S8. TEAE and TRAE leading to discontinuation of trastuzumab ........................................ 17
Table S9. TEAEs and TRAEs leading to discontinuation of docetaxel .......................................... 18
Table S10. TEAEs leading to administration delay of trastuzumab ............................................... 19
Table S11. TRAEs leading to administration delay of trastuzumab ............................................... 20
Table S12. TEAEs leading to administration delay of docetaxel .................................................... 21
Table S13. TRAEs leading to administration delay of docetaxel .................................................... 22
Table S14. TEAE and TRAE leading to infusion interruption of trastuzumab .............................. 23
Table S15. TEAEs and TRAEs leading to infusion interruption of docetaxel ................................ 24
Table S16. TEAEs and TRAEs leading to dose reduction of docetaxel .......................................... 25

Table S1. Summary of administration period and cumulative dose of study drugs (SAS)

| | | Total, N = 16 |
|---|---|---|
| Duration of trastuzumab administration (days) | Number of cases with data | 16 |
| | Mean | 170.8 |
| | STD | 24.4 |
| | Min | 84 |
| | median | 175.0 |
| | Max. | 192 |
| Duration of docetaxel administration (days) | Number of cases with data | 16 |
| | Mean | 162.9 |
| | STD | 35.8 |
| | Min | 63 |
| | median | 175.0 |
| | Max. | 192 |
| Cumulative dose of trastuzumab (mg/kg) | Number of cases with data | 16 |
| | Mean | 48.9 |
| | STD | 6.2 |
| | Min | 26 |
| | median | 50.0 |
| | Max. | 52 |
| Cumulative dose of docetaxel (mg/m$^2$) | Number of cases with data | 16 |
| | Mean | 485.9 |
| | STD | 103.9 |
| | Min | 210 |
| | median | 530.0 |
| | Max. | 560 |

Abbreviations: SAS, safety analysis set. Max, maximum value; STD, standard deviation; Min, minimum value.
N, number of subjects in the safety analysis set

Table S2. Summary of the number of doses, dose delays, interruptions, and reductions (SAS)

| Category | Classification/Reason | N = 16 | |
|---|---|---|---|
| | | n | (%) |
| Number of doses of trastuzumab | 1 | 0 | (0.0) |
| | 2 | 0 | (0.0) |
| | 3 | 0 | (0.0) |
| | 4 | 1 | (6.3) |
| | 5 | 0 | (0.0) |
| | 6 | 0 | (0.0) |
| | 7 | 0 | (0.0) |
| | 8 | 15 | (93.8) |
| Number of doses of docetaxel | 1 | 0 | (0.0) |
| | 2 | 0 | (0.0) |
| | 3 | 1 | (6.3) |
| | 4 | 1 | (6.3) |
| | 5 | 0 | (0.0) |
| | 6 | 0 | (0.0) |
| | 7 | 0 | (0.0) |
| | 8 | 14 | (87.5) |
| Dose delays | Total | 11 | (68.8) |
| | Trastuzumab administration criteria | 0 | (0.0) |
| | Docetaxel administration criteria | 1 | (6.3) |
| | Holidays, outpatient scheduling | 7 | (43.8) |
| | Other (including adverse events) | 6 | (37.5) |
| Infusion interruption of trastuzumab administration | Total | 1 | (6.3) |
| | Adverse events | 1 | (6.3) |
| Infusion interruption of docetaxel administration | Total | 1 | (6.3) |
| | Adverse events | 1 | (6.3) |
| Reduction of docetaxel dose | Total | 8 | (50.0) |
| | Adverse events | 8 | (50.0) |
| | Other | 1 | (6.3) |

Abbrivations: SAS, safety analysis set.

N is the number of patients in the safety analysis set. Data were presented as the number of patients. The percentage was calculated using N as the denominator.

Table S3. Subsequent treatment (SAS)

| SAS | N = 16 |
| --- | --- |
| | No. of patients |
| Subsequent treatment* | 12 |
| Before progression of protocol treatment | 4** |
| Systemic therapy | 4 |
| Trastuzumab + docetaxel as maintenance therapy | 2 |
| Docetaxel as maintenance therapy | 2 |
| After the progression of protocol treatment | 10 |
| Systemic therapy | 9 |
| Carboplatin + paclitaxel | 4 |
| Nivolumab | 3 |
| Cisplatin + 5-FU | 1 |
| S-1 | 1 |
| Paclitaxel + cetuximab | 1 |
| Bicalutamide + leuprorelin | 1 |
| Investigational drug | 4 |
| Intrathecal administration | 1 |
| Methotrexate + prednisolone | 1 |
| Surgery | 1 |
| Radiation | 2 |

Abbreviations: SAS, Safety analysis set; 5-FU, 5-fluorouracil; TS-1, tegafur/gimeracil/oteracil; FAS, full analysis set.
N is the number of subjects in the SAS.
*Subsequent treatment was not predefined. The patient excluded from full analysis set (FAS) did not receive subsequent systemic treatment.
**For these patients, PFS was censored at the date when the absence of progression was confirmed before starting the post-treatment.

Table S4. All TEAEs by worst grade

| [SOC] PT | Total, N = 16 | | Number of patients by worst grade | | | | | | |
|---|---|---|---|---|---|---|---|---|---|
| | n | (%) | G1 | G2 | G3 | G4 | G5 | G3 ≤ | G3 ≤ (%) |
| Any | 16 | (100.0) | 0 | 0 | 2 | 13 | 1 | 16 | (100.0) |
| [Blood and lymphatic system disorders] | 13 | (81.3) | 5 | 5 | 3 | 0 | 0 | 3 | (18.8) |
| Anemia | 13 | (81.3) | 6 | 6 | 1 | 0 | 0 | 1 | (6.3) |
| Febrile neutropenia | 2 | (12.5) | 0 | 0 | 2 | 0 | 0 | 2 | (12.5) |
| [Cardiac disorders] | 1 | (6.3) | 1 | 0 | 0 | 0 | 0 | 0 | (0.0) |
| Palpitations | 1 | (6.3) | 1 | 0 | 0 | 0 | 0 | 0 | (0.0) |
| [Eye disorders] | 4 | (25.0) | 3 | 1 | 0 | 0 | 0 | 0 | (0.0) |
| Dry eye | 1 | (6.3) | 0 | 1 | 0 | 0 | 0 | 0 | (0.0) |
| Lacrimation increased | 3 | (18.8) | 3 | 0 | 0 | 0 | 0 | 0 | (0.0) |
| [Gastrointestinal disorders] | 16 | (100.0) | 7 | 6 | 3 | 0 | 0 | 3 | (18.8) |
| Abdominal pain | 1 | (6.3) | 1 | 0 | 0 | 0 | 0 | 0 | (0.0) |
| Upper abdominal pain | 2 | (12.5) | 2 | 0 | 0 | 0 | 0 | 0 | (0.0) |
| Anal fistula | 1 | (6.3) | 0 | 0 | 1 | 0 | 0 | 1 | (6.3) |
| Constipation | 3 | (18.8) | 3 | 0 | 0 | 0 | 0 | 0 | (0.0) |
| Diarrhea | 4 | (25.0) | 2 | 2 | 0 | 0 | 0 | 0 | (0.0) |
| Dysphagia | 1 | (6.3) | 0 | 0 | 1 | 0 | 0 | 1 | (6.3) |
| Enteritis | 1 | (6.3) | 0 | 1 | 0 | 0 | 0 | 0 | (0.0) |
| Hemorrhoids | 1 | (6.3) | 0 | 1 | 0 | 0 | 0 | 0 | (0.0) |
| Nausea | 2 | (12.5) | 1 | 0 | 1 | 0 | 0 | 1 | (6.3) |
| Periodontal disease | 1 | (6.3) | 0 | 1 | 0 | 0 | 0 | 0 | (0.0) |
| Stomatitis | 10 | (62.5) | 6 | 4 | 0 | 0 | 0 | 0 | (0.0) |
| Upper gastrointestinal hemorrhage | 1 | (6.3) | 0 | 1 | 0 | 0 | 0 | 0 | (0.0) |
| Vomiting | 2 | (12.5) | 2 | 0 | 0 | 0 | 0 | 0 | (0.0) |
| [General disorders and administration site conditions] | 14 | (87.5) | 5 | 9 | 0 | 0 | 0 | 0 | (0.0) |
| Facial edema | 2 | (12.5) | 2 | 0 | 0 | 0 | 0 | 0 | (0.0) |

Abbreviations: TEAEs, treatment-emergent adverse events; SOC, system organ class; PT, preferred term; G, grade.

N is the number of patients in the safety analysis set. Data were presented as the number of patients. The percentage was calculated using N as the denominator. The adverse events recorded in the case report forms were coded, classified by SOC using MedDRA/J version 22.0, and were presented by PT.

Table S4. All TEAEs by worst grade

| [SOC] PT | Total, N = 16 n | (%) | Number of patients by worst grade G1 | G2 | G3 | G4 | G5 | G3 ≤ | G3 ≤ (%) |
|---|---|---|---|---|---|---|---|---|---|
| Fatigue | 2 | (12.5) | 0 | 2 | 0 | 0 | 0 | 0 | (0.0) |
| Malaise | 7 | (43.8) | 4 | 3 | 0 | 0 | 0 | 0 | (0.0) |
| Edema | 1 | (6.3) | 1 | 0 | 0 | 0 | 0 | 0 | (0.0) |
| Peripheral edema | 8 | (50.0) | 1 | 7 | 0 | 0 | 0 | 0 | (0.0) |
| Pain | 1 | (6.3) | 1 | 0 | 0 | 0 | 0 | 0 | (0.0) |
| Pyrexia | 4 | (25.0) | 3 | 1 | 0 | 0 | 0 | 0 | (0.0) |
| Localized edema | 1 | (6.3) | 1 | 0 | 0 | 0 | 0 | 0 | (0.0) |
| Non-cardiac chest pain | 1 | (6.3) | 1 | 0 | 0 | 0 | 0 | 0 | (0.0) |
| [Infections and infestations] | 8 | (50.0) | 3 | 4 | 1 | 0 | 0 | 1 | (6.3) |
| Bronchitis | 2 | (12.5) | 0 | 1 | 1 | 0 | 0 | 1 | (6.3) |
| Conjunctivitis | 1 | (6.3) | 0 | 1 | 0 | 0 | 0 | 0 | (0.0) |
| Gingivitis | 1 | (6.3) | 1 | 0 | 0 | 0 | 0 | 0 | (0.0) |
| Oral candidiasis | 1 | (6.3) | 0 | 1 | 0 | 0 | 0 | 0 | (0.0) |
| Otitis media | 1 | (6.3) | 1 | 0 | 0 | 0 | 0 | 0 | (0.0) |
| Paronychia | 2 | (12.5) | 1 | 1 | 0 | 0 | 0 | 0 | (0.0) |
| Periodontitis | 1 | (6.3) | 1 | 0 | 0 | 0 | 0 | 0 | (0.0) |
| Pharyngitis | 1 | (6.3) | 1 | 0 | 0 | 0 | 0 | 0 | (0.0) |
| Rhinitis | 1 | (6.3) | 0 | 1 | 0 | 0 | 0 | 0 | (0.0) |
| Sinusitis | 1 | (6.3) | 0 | 1 | 0 | 0 | 0 | 0 | (0.0) |
| Upper respiratory tract infection | 3 | (18.8) | 1 | 2 | 0 | 0 | 0 | 0 | (0.0) |
| Lung infection | 1 | (6.3) | 0 | 0 | 1 | 0 | 0 | 1 | (6.3) |
| [Injury, poisoning and procedural complications] | 5 | (31.3) | 2 | 3 | 0 | 0 | 0 | 0 | (0.0) |
| Infusion related reaction | 4 | (25.0) | 2 | 2 | 0 | 0 | 0 | 0 | (0.0) |
| Wound complication | 1 | (6.3) | 0 | 1 | 0 | 0 | 0 | 0 | (0.0) |
| [Investigations] | 16 | (100.0) | 0 | 0 | 2 | 14 | 0 | 16 | (100.0) |

Abbreviations: TEAEs, treatment-emergent adverse events; SOC, system organ class; PT, preferred term; G, grade.

N is the number of patients in the safety analysis set. Data were presented as the number of patients. The percentage was calculated using N as the denominator. The adverse events recorded in the case report forms were coded, classified by SOC using MedDRA/J version 22.0, and were presented by PT.

Table S4. All TEAEs by worst grade

| [SOC] PT | Total, N = 16 n | (%) | Number of patients by worst grade G1 | G2 | G3 | G4 | G5 | G3 ≤ | G3 ≤ (%) |
|---|---|---|---|---|---|---|---|---|---|
| Increased alanine aminotransferase | 5 | (31.3) | 4 | 1 | 0 | 0 | 0 | 0 | (0.0) |
| Increased aspartate aminotransferase | 1 | (6.3) | 1 | 0 | 0 | 0 | 0 | 0 | (0.0) |
| Increased blood creatinine | 1 | (6.3) | 1 | 0 | 0 | 0 | 0 | 0 | (0.0) |
| Increased blood lactate dehydrogenase | 5 | (31.3) | 5 | 0 | 0 | 0 | 0 | 0 | (0.0) |
| Increased gamma-glutamyltransferase | 1 | (6.3) | 1 | 0 | 0 | 0 | 0 | 0 | (0.0) |
| Lymphocyte count decreased | 5 | (31.3) | 0 | 2 | 3 | 0 | 0 | 3 | (18.8) |
| Neutrophil count decreased | 16 | (100.0) | 0 | 0 | 2 | 14 | 0 | 16 | (100.0) |
| Hypoproteinemia | 1 | (6.3) | 1 | 0 | 0 | 0 | 0 | 0 | (0.0) |
| Weight decreased | 1 | (6.3) | 0 | 1 | 0 | 0 | 0 | 0 | (0.0) |
| Weight increased | 6 | (37.5) | 3 | 3 | 0 | 0 | 0 | 0 | (0.0) |
| White blood cell count decreased | 15 | (93.8) | 0 | 0 | 10 | 5 | 0 | 15 | (93.8) |
| Urine output decreased | 1 | (6.3) | 0 | 0 | 1 | 0 | 0 | 1 | (6.3) |
| [Metabolism and nutrition disorders] | 14 | (87.5) | 8 | 3 | 2 | 0 | 1 | 3 | (18.8) |
| Hyperglycemia | 1 | (6.3) | 0 | 0 | 1 | 0 | 0 | 1 | (6.3) |
| Hyperkalemia | 2 | (12.5) | 1 | 1 | 0 | 0 | 0 | 0 | (0.0) |
| Hyperuricemia | 1 | (6.3) | 1 | 0 | 0 | 0 | 0 | 0 | (0.0) |
| Hypoalbuminemia | 10 | (62.5) | 6 | 3 | 0 | 0 | 1 | 1 | (6.3) |
| Hypocalcemia | 4 | (25.0) | 4 | 0 | 0 | 0 | 0 | 0 | (0.0) |
| Hypokalemia | 1 | (6.3) | 0 | 0 | 1 | 0 | 0 | 1 | (6.3) |
| Hyponatremia | 2 | (12.5) | 2 | 0 | 0 | 0 | 0 | 0 | (0.0) |
| Hypophosphatemia | 1 | (6.3) | 0 | 1 | 0 | 0 | 0 | 0 | (0.0) |

Abbreviations: TEAEs, treatment-emergent adverse events; SOC, system organ class; PT, preferred term; G, grade.

N is the number of patients in the safety analysis set. Data were presented as the number of patients. The percentage was calculated using N as the denominator. The adverse events recorded in the case report forms were coded, classified by SOC using MedDRA/J version 22.0, and were presented by PT.

Table S4. All TEAEs by worst grade

| [SOC] | Total, N = 16 | | Number of patients by worst grade | | | | | | |
|---|---|---|---|---|---|---|---|---|---|
| PT | n | (%) | G1 | G2 | G3 | G4 | G5 | G3 ≤ | G3 ≤ (%) |
| Decreased appetite | 7 | (43.8) | 5 | 1 | 1 | 0 | 0 | 1 | (6.3) |
| [Musculoskeletal and connective tissue disorders] | 5 | (31.3) | 3 | 2 | 0 | 0 | 0 | 0 | (0.0) |
| Arthralgia | 2 | (12.5) | 1 | 1 | 0 | 0 | 0 | 0 | (0.0) |
| Back pain | 3 | (18.8) | 3 | 0 | 0 | 0 | 0 | 0 | (0.0) |
| Musculoskeletal pain | 2 | (12.5) | 1 | 1 | 0 | 0 | 0 | 0 | (0.0) |
| Myalgia | 2 | (12.5) | 1 | 1 | 0 | 0 | 0 | 0 | (0.0) |
| [Nervous system disorders] | 7 | (43.8) | 4 | 3 | 0 | 0 | 0 | 0 | (0.0) |
| Dysgeusia | 1 | (6.3) | 0 | 1 | 0 | 0 | 0 | 0 | (0.0) |
| Head discomfort | 1 | (6.3) | 1 | 0 | 0 | 0 | 0 | 0 | (0.0) |
| Headache | 1 | (6.3) | 1 | 0 | 0 | 0 | 0 | 0 | (0.0) |
| Peripheral neuropathy | 1 | (6.3) | 0 | 1 | 0 | 0 | 0 | 0 | (0.0) |
| Peripheral sensory neuropathy | 4 | (25.0) | 3 | 1 | 0 | 0 | 0 | 0 | (0.0) |
| Taste disorder | 1 | (6.3) | 1 | 0 | 0 | 0 | 0 | 0 | (0.0) |
| [Psychiatric disorders] | 1 | (6.3) | 0 | 0 | 1 | 0 | 0 | 1 | (6.3) |
| Delirium | 1 | (6.3) | 0 | 1 | 0 | 0 | 0 | 0 | (0.0) |
| Insomnia | 1 | (6.3) | 0 | 0 | 1 | 0 | 0 | 1 | (6.3) |
| [Renal and urinary disorders] | 1 | (6.3) | 1 | 0 | 0 | 0 | 0 | 0 | (0.0) |
| Proteinuria | 1 | (6.3) | 1 | 0 | 0 | 0 | 0 | 0 | (0.0) |
| [Reproductive system and breast disorders] | 1 | (6.3) | 1 | 0 | 0 | 0 | 0 | 0 | (0.0) |
| Irregular menstruation | 1 | (6.3) | 1 | 0 | 0 | 0 | 0 | 0 | (0.0) |
| [Respiratory, thoracic and mediastinal disorders] | 12 | (75.0) | 8 | 3 | 1 | 0 | 0 | 1 | (6.3) |
| Cough | 4 | (25.0) | 4 | 0 | 0 | 0 | 0 | 0 | (0.0) |
| Dyspnea | 2 | (12.5) | 1 | 1 | 0 | 0 | 0 | 0 | (0.0) |
| Epistaxis | 2 | (12.5) | 2 | 0 | 0 | 0 | 0 | 0 | (0.0) |

Abbreviations: TEAEs, treatment-emergent adverse events; SOC, system organ class; PT, preferred term; G, grade.

N is the number of patients in the safety analysis set. Data were presented as the number of patients. The percentage was calculated using N as the denominator. The adverse events recorded in the case report forms were coded, classified by SOC using MedDRA/J version 22.0, and were presented by PT.

Table S4. All TEAEs by worst grade

| [SOC]<br>PT | Total, N = 16 n | (%) | Number of patients by worst grade G1 | G2 | G3 | G4 | G5 | G3 ≤ | G3 ≤ (%) |
|---|---|---|---|---|---|---|---|---|---|
| Hiccups | 1 | (6.3) | 1 | 0 | 0 | 0 | 0 | 0 | (0.0) |
| Hypoxia | 1 | (6.3) | 0 | 1 | 0 | 0 | 0 | 0 | (0.0) |
| Pleural effusion | 5 | (31.3) | 3 | 2 | 0 | 0 | 0 | 0 | (0.0) |
| Aspiration pneumonia | 1 | (6.3) | 0 | 0 | 1 | 0 | 0 | 1 | (6.3) |
| Pneumonitis | 1 | (6.3) | 1 | 0 | 0 | 0 | 0 | 0 | (0.0) |
| Allergic rhinitis | 1 | (6.3) | 0 | 1 | 0 | 0 | 0 | 0 | (0.0) |
| Oropharyngeal pain | 1 | (6.3) | 1 | 0 | 0 | 0 | 0 | 0 | (0.0) |
| [Skin and subcutaneous tissue disorders] | 16 | (100.0) | 7 | 9 | 0 | 0 | 0 | 0 | (0.0) |
| Alopecia | 14 | (87.5) | 8 | 6 | 0 | 0 | 0 | 0 | (0.0) |
| Pressure ulcer | 1 | (6.3) | 1 | 0 | 0 | 0 | 0 | 0 | (0.0) |
| Dermatitis | 1 | (6.3) | 0 | 1 | 0 | 0 | 0 | 0 | (0.0) |
| Acneiform dermatitis | 1 | (6.3) | 1 | 0 | 0 | 0 | 0 | 0 | (0.0) |
| Contact dermatitis | 1 | (6.3) | 0 | 1 | 0 | 0 | 0 | 0 | (0.0) |
| Dry skin | 3 | (18.8) | 3 | 0 | 0 | 0 | 0 | 0 | (0.0) |
| Eczema | 2 | (12.5) | 1 | 1 | 0 | 0 | 0 | 0 | (0.0) |
| Erythema | 1 | (6.3) | 0 | 1 | 0 | 0 | 0 | 0 | (0.0) |
| Nail discoloration | 1 | (6.3) | 1 | 0 | 0 | 0 | 0 | 0 | (0.0) |
| Nail disorder | 3 | (18.8) | 3 | 0 | 0 | 0 | 0 | 0 | (0.0) |
| Palmar-plantar erythrodysesthesia syndrome | 1 | (6.3) | 1 | 0 | 0 | 0 | 0 | 0 | (0.0) |
| Pruritus | 2 | (12.5) | 2 | 0 | 0 | 0 | 0 | 0 | (0.0) |
| Rash | 2 | (12.5) | 1 | 1 | 0 | 0 | 0 | 0 | (0.0) |
| Skin fissures | 1 | (6.3) | 1 | 0 | 0 | 0 | 0 | 0 | (0.0) |
| Onycholysis | 1 | (6.3) | 0 | 1 | 0 | 0 | 0 | 0 | (0.0) |
| Pigmentation disorder | 1 | (6.3) | 1 | 0 | 0 | 0 | 0 | 0 | (0.0) |
| Nail ridging | 3 | (18.8) | 3 | 0 | 0 | 0 | 0 | 0 | (0.0) |
| [Vascular disorders] | 3 | (18.8) | 1 | 2 | 0 | 0 | 0 | 0 | (0.0) |

Abbreviations: TEAEs, treatment-emergent adverse events; SOC, system organ class; PT, preferred term; G, grade.

N is the number of patients in the safety analysis set. Data were presented as the number of patients. The percentage was calculated using N as the denominator. The adverse events recorded in the case report forms were coded, classified by SOC using MedDRA/J version 22.0, and were presented by PT.

Table S4. All TEAEs by worst grade

| [SOC] | Total, N = 16 | | Number of patients by worst grade | | | | | | |
|---|---|---|---|---|---|---|---|---|---|
| PT | n | (%) | G1 | G2 | G3 | G4 | G5 | G3 ≤ | G3 ≤ (%) |
| Hypertension | 1 | (6.3) | 0 | 1 | 0 | 0 | 0 | 0 | (0.0) |
| Thrombosis | 1 | (6.3) | 0 | 1 | 0 | 0 | 0 | 0 | (0.0) |
| Vasculitis | 1 | (6.3) | 1 | 0 | 0 | 0 | 0 | 0 | (0.0) |

Abbreviations: TEAEs, treatment-emergent adverse events; SOC, system organ class; PT, preferred term; G, grade.

N is the number of patients in the safety analysis set. Data were presented as the number of patients. The percentage was calculated using N as the denominator. The adverse events recorded in the case report forms were coded, classified by SOC using MedDRA/J version 22.0, and were presented by PT.

Table S5. All TRAEs by worst grade

| [SOC]<br>PT | Total, N = 16 | | Number of subjects by worst grade | | | | | | |
|---|---|---|---|---|---|---|---|---|---|
| | n | (%) | G1 | G2 | G3 | G4 | G5 | G3 ≤ | G3 ≤ (%) |
| Any | 16 | (100.0) | 0 | 0 | 2 | 13 | 1 | 16 | (100.0) |
| [Blood and lymphatic system disorders] | 13 | (81.3) | 5 | 5 | 3 | 0 | 0 | 3 | (18.8) |
| Anemia | 13 | (81.3) | 6 | 6 | 1 | 0 | 0 | 1 | (6.3) |
| Febrile neutropenia | 2 | (12.5) | 0 | 0 | 2 | 0 | 0 | 2 | (12.5) |
| [Cardiac disorders] | 1 | (6.3) | 1 | 0 | 0 | 0 | 0 | 0 | (0.0) |
| Palpitations | 1 | (6.3) | 1 | 0 | 0 | 0 | 0 | 0 | (0.0) |
| [Eye disorders] | 3 | (18.8) | 3 | 0 | 0 | 0 | 0 | 0 | (0.0) |
| Lacrimation increased | 3 | (18.8) | 3 | 0 | 0 | 0 | 0 | 0 | (0.0) |
| [Gastrointestinal disorders] | 13 | (81.3) | 7 | 5 | 1 | 0 | 0 | 1 | (6.3) |
| Abdominal pain | 1 | (6.3) | 1 | 0 | 0 | 0 | 0 | 0 | (0.0) |
| Upper abdominal pain | 2 | (12.5) | 2 | 0 | 0 | 0 | 0 | 0 | (0.0) |
| Constipation | 1 | (6.3) | 1 | 0 | 0 | 0 | 0 | 0 | (0.0) |
| Diarrhea | 4 | (25.0) | 2 | 2 | 0 | 0 | 0 | 0 | (0.0) |
| Hemorrhoids | 1 | (6.3) | 0 | 1 | 0 | 0 | 0 | 0 | (0.0) |
| Nausea | 2 | (12.5) | 1 | 0 | 1 | 0 | 0 | 1 | (6.3) |
| Periodontal disease | 1 | (6.3) | 0 | 1 | 0 | 0 | 0 | 0 | (0.0) |
| Stomatitis | 10 | (62.5) | 6 | 4 | 0 | 0 | 0 | 0 | (0.0) |
| Upper gastrointestinal hemorrhage | 1 | (6.3) | 0 | 1 | 0 | 0 | 0 | 0 | (0.0) |
| Vomiting | 2 | (12.5) | 2 | 0 | 0 | 0 | 0 | 0 | (0.0) |
| [General disorders and administration site conditions] | 13 | (81.3) | 4 | 9 | 0 | 0 | 0 | 0 | (0.0) |
| Facial edema | 2 | (12.5) | 2 | 0 | 0 | 0 | 0 | 0 | (0.0) |
| Fatigue | 2 | (12.5) | 0 | 2 | 0 | 0 | 0 | 0 | (0.0) |
| Malaise | 6 | (37.5) | 3 | 3 | 0 | 0 | 0 | 0 | (0.0) |
| Edema | 1 | (6.3) | 1 | 0 | 0 | 0 | 0 | 0 | (0.0) |
| Peripheral edema | 8 | (50.0) | 1 | 7 | 0 | 0 | 0 | 0 | (0.0) |
| Pain | 1 | (6.3) | 1 | 0 | 0 | 0 | 0 | 0 | (0.0) |
| Pyrexia | 2 | (12.5) | 1 | 1 | 0 | 0 | 0 | 0 | (0.0) |

Abbreviations: TRAEs, treatment-related adverse events; SOC, system organ class; PT, preferred term; G, grade.
N is the number of patients in the safety analysis set. Data were presented as the number of patients. The percentage was calculated using N as the denominator. The adverse events recorded in the case report forms were coded, classified by SOC using MedDRA/J version 22.0, and were presented by PT.

Table S5. All TRAEs by worst grade

| [SOC]<br>PT | Total, N = 16 n | (%) | Number of subjects by worst grade G1 | G2 | G3 | G4 | G5 | G3 ≤ | G3 ≤ (%) |
|---|---|---|---|---|---|---|---|---|---|
| Localized edema | 1 | (6.3) | 1 | 0 | 0 | 0 | 0 | 0 | (0.0) |
| [Infections and infestations] | 3 | (18.8) | 2 | 0 | 1 | 0 | 0 | 1 | (6.3) |
| Bronchitis | 2 | (12.5) | 0 | 1 | 1 | 0 | 0 | 1 | (6.3) |
| Paronychia | 1 | (6.3) | 1 | 0 | 0 | 0 | 0 | 0 | (0.0) |
| Periodontitis | 1 | (6.3) | 0 | 1 | 0 | 0 | 0 | 0 | (0.0) |
| Rhinitis | 1 | (6.3) | 0 | 1 | 0 | 0 | 0 | 0 | (0.0) |
| Lung infection | 1 | (6.3) | 0 | 0 | 1 | 0 | 0 | 1 | (6.3) |
| [Injury, poisoning and procedural complications] | 4 | (25.0) | 2 | 2 | 0 | 0 | 0 | 0 | (0.0) |
| Infusion related reaction | 4 | (25.0) | 2 | 2 | 0 | 0 | 0 | 0 | (0.0) |
| [Investigations] | 16 | (100.0) | 0 | 0 | 2 | 14 | 0 | 16 | (100.0) |
| Increased alanine aminotransferase | 5 | (31.3) | 4 | 1 | 0 | 0 | 0 | 0 | (0.0) |
| Increased aspartate aminotransferase | 1 | (6.3) | 1 | 0 | 0 | 0 | 0 | 0 | (0.0) |
| Increased blood creatinine | 1 | (6.3) | 1 | 0 | 0 | 0 | 0 | 0 | (0.0) |
| Increased blood lactate dehydrogenase | 2 | (12.5) | 2 | 0 | 0 | 0 | 0 | 0 | (0.0) |
| Lymphocyte count decreased | 5 | (31.3) | 0 | 2 | 3 | 0 | 0 | 3 | (18.8) |
| Neutrophil count decreased | 16 | (100.0) | 0 | 0 | 2 | 14 | 0 | 16 | (100.0) |
| Hypoproteinemia | 1 | (6.3) | 1 | 0 | 0 | 0 | 0 | 0 | (0.0) |
| Weight decreased | 1 | (6.3) | 0 | 1 | 0 | 0 | 0 | 0 | (0.0) |
| Weight increased | 4 | (25.0) | 2 | 2 | 0 | 0 | 0 | 0 | (0.0) |
| White blood cell count decreased | 15 | (93.8) | 0 | 0 | 10 | 5 | 0 | 15 | (93.8) |
| [Metabolism and nutrition disorders] | 11 | (68.8) | 7 | 3 | 0 | 0 | 1 | 1 | (6.3) |
| Hyperkalemia | 1 | (6.3) | 0 | 1 | 0 | 0 | 0 | 0 | (0.0) |
| Hypoalbuminemia | 9 | (56.3) | 5 | 3 | 0 | 0 | 1 | 1 | (6.3) |

Abbreviations: TRAEs, treatment-related adverse events; SOC, system organ class; PT, preferred term; G, grade.
N is the number of patients in the safety analysis set. Data were presented as the number of patients. The percentage was calculated using N as the denominator. The adverse events recorded in the case report forms were coded, classified by SOC using MedDRA/J version 22.0, and were presented by PT.

Table S5. All TRAEs by worst grade

| [SOC] PT | Total, N = 16 n | (%) | Number of subjects by worst grade G1 | G2 | G3 | G4 | G5 | G3 ≤ | G3 ≤ (%) |
|---|---|---|---|---|---|---|---|---|---|
| Hypocalcemia | 3 | (18.8) | 3 | 0 | 0 | 0 | 0 | 0 | (0.0) |
| Hyponatremia | 1 | (6.3) | 1 | 0 | 0 | 0 | 0 | 0 | (0.0) |
| Hypophosphatemia | 1 | (6.3) | 0 | 1 | 0 | 0 | 0 | 0 | (0.0) |
| Decreased appetite | 7 | (43.8) | 5 | 1 | 1 | 0 | 0 | 1 | (6.3) |
| [Musculoskeletal and connective tissue disorders] | 2 | (12.5) | 1 | 1 | 0 | 0 | 0 | 0 | (0.0) |
| Arthralgia | 1 | (6.3) | 0 | 1 | 0 | 0 | 0 | 0 | (0.0) |
| Myalgia | 2 | (12.5) | 1 | 1 | 0 | 0 | 0 | 0 | (0.0) |
| [Nervous system disorders] | 7 | (43.8) | 4 | 3 | 0 | 0 | 0 | 0 | (0.0) |
| Dysgeusia | 1 | (6.3) | 1 | 0 | 0 | 0 | 0 | 0 | (0.0) |
| Head discomfort | 1 | (6.3) | 1 | 0 | 0 | 0 | 0 | 0 | (0.0) |
| Headache | 1 | (6.3) | 1 | 0 | 0 | 0 | 0 | 0 | (0.0) |
| Peripheral neuropathy | 1 | (6.3) | 0 | 1 | 0 | 0 | 0 | 0 | (0.0) |
| Peripheral sensory neuropathy | 4 | (25.0) | 3 | 1 | 0 | 0 | 0 | 0 | (0.0) |
| Taste disorder | 1 | (6.3) | 0 | 1 | 0 | 0 | 0 | 0 | (0.0) |
| [Psychiatric disorders] | 1 | (6.3) | 0 | 0 | 1 | 0 | 0 | 1 | (6.3) |
| Insomnia | 1 | (6.3) | 0 | 0 | 1 | 0 | 0 | 1 | (6.3) |
| [Renal and urinary disorders] | 1 | (6.3) | 1 | 0 | 0 | 0 | 0 | 0 | (0.0) |
| Proteinuria | 1 | (6.3) | 1 | 0 | 0 | 0 | 0 | 0 | (0.0) |
| [Reproductive system and breast disorders] | 1 | (6.3) | 1 | 0 | 0 | 0 | 0 | 0 | (0.0) |
| Irregular menstruation | 1 | (6.3) | 1 | 0 | 0 | 0 | 0 | 0 | (0.0) |
| [Respiratory, thoracic and mediastinal disorders] | 7 | (43.8) | 5 | 2 | 0 | 0 | 0 | 0 | (0.0) |
| Cough | 2 | (12.5) | 2 | 0 | 0 | 0 | 0 | 0 | (0.0) |
| Dyspnea | 2 | (12.5) | 1 | 1 | 0 | 0 | 0 | 0 | (0.0) |
| Epistaxis | 1 | (6.3) | 1 | 0 | 0 | 0 | 0 | 0 | (0.0) |
| Hiccups | 1 | (6.3) | 1 | 0 | 0 | 0 | 0 | 0 | (0.0) |
| Pleural effusion | 5 | (31.3) | 3 | 2 | 0 | 0 | 0 | 0 | (0.0) |

Abbreviations: TRAEs, treatment-related adverse events; SOC, system organ class; PT, preferred term; G, grade.
N is the number of patients in the safety analysis set. Data were presented as the number of patients. The percentage was calculated using N as the denominator. The adverse events recorded in the case report forms were coded, classified by SOC using MedDRA/J version 22.0, and were presented by PT.

Table S5. All TRAEs by worst grade

| [SOC]<br>PT | Total, N = 16 n | (%) | Number of subjects by worst grade G1 | G2 | G3 | G4 | G5 | G3 ≤ | G3 ≤ (%) |
|---|---|---|---|---|---|---|---|---|---|
| Pneumonitis | 1 | (6.3) | 1 | 0 | 0 | 0 | 0 | 0 | (0.0) |
| Oropharyngeal pain | 1 | (6.3) | 1 | 0 | 0 | 0 | 0 | 0 | (0.0) |
| [Skin and subcutaneous tissue disorders] | 10 | (62.5) | 4 | 6 | 0 | 0 | 0 | 0 | (0.0) |
| Alopecia | 14 | (87.5) | 8 | 6 | 0 | 0 | 0 | 0 | (0.0) |
| Dry skin | 3 | (18.8) | 3 | 0 | 0 | 0 | 0 | 0 | (0.0) |
| Eczema | 1 | (6.3) | 0 | 1 | 0 | 0 | 0 | 0 | (0.0) |
| Nail discoloration | 1 | (6.3) | 1 | 0 | 0 | 0 | 0 | 0 | (0.0) |
| Nail disorder | 3 | (18.8) | 3 | 0 | 0 | 0 | 0 | 0 | (0.0) |
| Palmar-plantar erythrodysesthesia syndrome | 1 | (6.3) | 1 | 0 | 0 | 0 | 0 | 0 | (0.0) |
| Pruritus | 1 | (6.3) | 1 | 0 | 0 | 0 | 0 | 0 | (0.0) |
| Rash | 1 | (6.3) | 1 | 0 | 0 | 0 | 0 | 0 | (0.0) |
| Skin fissures | 1 | (6.3) | 1 | 0 | 0 | 0 | 0 | 0 | (0.0) |
| Onycholysis | 1 | (6.3) | 0 | 1 | 0 | 0 | 0 | 0 | (0.0) |
| Pigmentation disorder | 1 | (6.3) | 1 | 0 | 0 | 0 | 0 | 0 | (0.0) |
| Nail ridging | 3 | (18.8) | 3 | 0 | 0 | 0 | 0 | 0 | (0.0) |
| [Vascular disorders] | 2 | (12.5) | 1 | 1 | 0 | 0 | 0 | 0 | (0.0) |
| Hypertension | 1 | (6.3) | 0 | 1 | 0 | 0 | 0 | 0 | (0.0) |
| Vasculitis | 1 | (6.3) | 1 | 0 | 0 | 0 | 0 | 0 | (0.0) |

Abbreviations: TRAEs, treatment-related adverse events; SOC, system organ class; PT, preferred term; G, grade.
N is the number of patients in the safety analysis set. Data were presented as the number of patients. The percentage was calculated using N as the denominator. The adverse events recorded in the case report forms were coded, classified by SOC using MedDRA/J version 22.0, and were presented by PT.

Table S6. Treatment-emergent SAEs by worst grade

| [SOC] | Total, N=16 | | Number of subjects by worst grade | | | | | | |
|---|---|---|---|---|---|---|---|---|---|
| PT | n | (%) | G1 | G2 | G3 | G4 | G5 | G3 ≤ | G3 ≤ (%) |
| Any | 7 | (43.8) | 0 | 1 | 2 | 3 | 1 | 6 | (37.5) |
| [Blood and lymphatic system disorders] | 2 | (12.5) | 0 | 0 | 2 | 0 | 0 | 2 | (12.5) |
| Anemia | 1 | (6.3) | 0 | 0 | 1 | 0 | 0 | 1 | (6.3) |
| Febrile neutropenia | 1 | (6.3) | 0 | 0 | 1 | 0 | 0 | 1 | (6.3) |
| [Gastrointestinal disorders] | 1 | (6.3) | 0 | 0 | 1 | 0 | 0 | 1 | (6.3) |
| Dysphagia | 1 | (6.3) | 0 | 0 | 1 | 0 | 0 | 1 | (6.3) |
| [General disorders and administration site conditions] | 2 | (12.5) | 0 | 2 | 0 | 0 | 0 | 0 | (0.0) |
| Malaise | 1 | (6.3) | 0 | 1 | 0 | 0 | 0 | 0 | (0.0) |
| Pyrexia | 1 | (6.3) | 0 | 1 | 0 | 0 | 0 | 0 | (0.0) |
| [Infections and infestations] | 1 | (6.3) | 0 | 0 | 1 | 0 | 0 | 1 | (6.3) |
| Bronchitis | 1 | (6.3) | 0 | 0 | 1 | 0 | 0 | 1 | (6.3) |
| Lung infection | 1 | (6.3) | 0 | 0 | 1 | 0 | 0 | 1 | (6.3) |
| [Investigations] | 3 | (18.8) | 0 | 0 | 0 | 3 | 0 | 3 | (18.8) |
| Neutropenia | 3 | (18.8) | 0 | 0 | 0 | 3 | 0 | 3 | (18.8) |
| [Metabolism and nutrition disorders] | 1 | (6.3) | 0 | 0 | 1 | 0 | 0 | 1 | (6.3) |
| Decreased appetite | 1 | (6.3) | 0 | 0 | 1 | 0 | 0 | 1 | (6.3) |
| Hypoalbuminemia | 1 | (6.3) | 0 | 0 | 0 | 0 | 1 | 1 | (6.3) |
| [Respiratory, thoracic and mediastinal disorders] | 1 | (6.3) | 0 | 0 | 1 | 0 | 0 | 1 | (6.3) |
| Aspiration pneumonia | 1 | (6.3) | 0 | 0 | 1 | 0 | 0 | 1 | (6.3) |

Abbreviations: SAEs, serious adverse events; SOC, system organ class; PT, preferred term; G, grade.
N is the number of patients in the safety analysis set. Data were presented as the number of patients. The percentage was calculated using N as the denominator. The adverse events recorded in the case report forms were coded, classified by SOC using MedDRA/J version 22.0, and were presented by PT.

Table S7. Treatment-related SAEs by worst grade

| [SOC] | Total, N=16 | | Number of subjects by worst grade | | | | | | |
|---|---|---|---|---|---|---|---|---|---|
| PT | n | (%) | G1 | G2 | G3 | G4 | G5 | G3 ≤ | G3 ≤ (%) |
| Any | 7 | (43.8) | 0 | 1 | 2 | 3 | 1 | 6 | (37.5) |
| [Blood and lymphatic system disorders] | 2 | (12.5) | 0 | 0 | 2 | 0 | 0 | 2 | (12.5) |
| Anemia | 1 | (6.3) | 0 | 0 | 1 | 0 | 0 | 1 | (6.3) |
| Febrile neutropenia | 1 | (6.3) | 0 | 0 | 1 | 0 | 0 | 1 | (6.3) |
| [General disorders and administration site conditions] | 2 | (12.5) | 0 | 2 | 0 | 0 | 0 | 0 | (0.0) |
| Malaise | 1 | (6.3) | 0 | 1 | 0 | 0 | 0 | 0 | (0.0) |
| Pyrexia | 1 | (6.3) | 0 | 1 | 0 | 0 | 0 | 0 | (0.0) |
| [Infections and infestations] | 1 | (6.3) | 0 | 0 | 1 | 0 | 0 | 1 | (6.3) |
| Bronchitis | 1 | (6.3) | 0 | 0 | 1 | 0 | 0 | 1 | (6.3) |
| Lung infection | 1 | (6.3) | 0 | 0 | 1 | 0 | 0 | 1 | (6.3) |
| [Investigations] | 3 | (18.8) | 0 | 0 | 0 | 3 | 0 | 3 | (18.8) |
| Neutropenia | 3 | (18.8) | 0 | 0 | 0 | 3 | 0 | 3 | (18.8) |
| [Metabolism and nutrition disorders] | 1 | (6.3) | 0 | 0 | 1 | 0 | 0 | 1 | (6.3) |
| Decreased appetite | 1 | (6.3) | 0 | 0 | 1 | 0 | 0 | 1 | (6.3) |
| Hypoalbuminemia | 1 | (6.3) | 0 | 0 | 0 | 0 | 1 | 1 | (6.3) |

Abbreviations: SAEs, serious adverse events; SOC, system organ class; PT, preferred term; G, grade.
N is the number of patients in the safety analysis set. Data were presented as the number of patients. The percentage was calculated using N as the denominator. The adverse events recorded in the case report forms were coded, classified by SOC using MedDRA/J version 22.0, and were presented by PT.

Table S8. TEAE and TRAE leading to discontinuation of trastuzumab

| [SOC] | Total, N=16 | | Number of Subjects by Worst Grade | | | | | | |
|---|---|---|---|---|---|---|---|---|---|
| PT | n | (%) | G1 | G2 | G3 | G4 | G5 | G3 ≤ | G3 ≤ (%) |
| Any | 1 | (6.3) | 1 | 0 | 0 | 0 | 1 | 1 | (6.3) |
| [Metabolism and nutrition disorders] | 1 | (6.3) | 1 | 0 | 0 | 0 | 1 | 1 | (6.3) |
| Hypoalbuminemia | 1 | (6.3) | 1 | 0 | 0 | 0 | 1 | 1 | (6.3) |

Abbreviations: TEAEs, treatment-emergent adverse events; TRAEs, treatment-related adverse events; SOC, system organ class; PT, preferred term; G, grade. N is the number of patients in the safety analysis set. Data were presented as the number of patients. The percentage was calculated using N as the denominator. The adverse events recorded in the case report forms were coded, classified by SOC using MedDRA/J version 22.0, and were presented by PT.

Table S9. TEAEs and TRAEs leading to discontinuation of docetaxel

| [SOC] | Total, N=16 | | Number of Subjects by Worst Grade | | | | | | |
|---|---|---|---|---|---|---|---|---|---|
| PT | n | (%) | G1 | G2 | G3 | G4 | G5 | G3 ≤ | G3 ≤ (%) |
| Any | 2 | (12.5) | 1 | 0 | 0 | 0 | 1 | 1 | (6.3) |
| [Metabolism and nutrition disorders] | 1 | (6.3) | 1 | 0 | 0 | 0 | 1 | 1 | (6.3) |
| Hypoalbuminemia | 1 | (6.3) | 1 | 0 | 0 | 0 | 1 | 1 | (6.3) |
| [Respiratory, thoracic and mediastinal disorders] | 1 | (6.3) | 1 | 0 | 0 | 0 | 0 | 0 | (0.0) |
| Pneumonitis | 1 | (6.3) | 1 | 0 | 0 | 0 | 0 | 0 | (0.0) |

Abbreviations: TEAEs, treatment-emergent adverse events; TRAEs, treatment-related adverse events; SOC, system organ class; PT, preferred term; G, grade. N is the number of patients in the safety analysis set. Data were presented as the number of patients. The percentage was calculated using N as the denominator. The adverse events recorded in the case report forms were coded, classified by SOC using MedDRA/J version 22.0, and were presented by PT.

Table S10. TEAEs leading to administration delay of trastuzumab

| [SOC] PT | Total, N = 16 n | (%) | Number of subjects by worst grade G1 | G2 | G3 | G4 | G5 | G3 ≤ | G3 ≤ (%) |
|---|---|---|---|---|---|---|---|---|---|
| Any | 7 | (43.8) | 1 | 4 | 1 | 1 | 0 | 2 | (12.5) |
| [Gastrointestinal disorders] | 1 | (6.3) | 0 | 1 | 0 | 0 | 0 | 0 | (0.0) |
| Periodontal disease | 1 | (6.3) | 0 | 1 | 0 | 0 | 0 | 0 | (0.0) |
| [General disorders and administration site conditions] | 1 | (6.3) | 0 | 1 | 0 | 0 | 0 | 0 | (0.0) |
| Pyrexia | 1 | (6.3) | 0 | 1 | 0 | 0 | 0 | 0 | (0.0) |
| [Infections and infestations] | 2 | (12.5) | 0 | 1 | 1 | 0 | 0 | 0 | (0.0) |
| Bronchitis | 1 | (6.3) | 0 | 0 | 1 | 0 | 0 | 1 | (6.3) |
| Upper respiratory tract infection | 1 | (6.3) | 0 | 1 | 0 | 0 | 0 | 0 | (0.0) |
| Lung infection | 1 | (6.3) | 0 | 0 | 1 | 0 | 0 | 1 | (6.3) |
| [Investigations] | 1 | (6.3) | 0 | 0 | 0 | 1 | 0 | 1 | (6.3) |
| Neutrophil count decreased | 1 | (6.3) | 0 | 0 | 0 | 1 | 0 | 1 | (6.3) |
| [Respiratory, thoracic and mediastinal disorders | 2 | (12.5) | 1 | 1 | 0 | 0 | 0 | 0 | (0.0) |
| Dyspnea | 1 | (6.3) | 0 | 1 | 0 | 0 | 0 | 0 | (0.0) |
| Pleural effusion | 1 | (6.3) | 0 | 1 | 0 | 0 | 0 | 0 | (0.0) |
| Pneumonitis | 1 | (6.3) | 1 | 0 | 0 | 0 | 0 | 0 | (0.0) |

Abbreviations: TEAEs, treatment-emergent adverse events; SOC, system organ class; PT, preferred term; G, grade.
N is the number of patients in the safety analysis set. Data were presented as the number of patients. The percentage was calculated using N as the denominator. The adverse events recorded in the case report forms were coded, classified by SOC using MedDRA/J version 22.0, and were presented by PT.

Table S11. TRAEs leading to administration delay of trastuzumab

| [SOC] | Total, N = 16 | | Number of patients by worst grade | | | | | | |
|---|---|---|---|---|---|---|---|---|---|
| PT | n | (%) | G1 | G2 | G3 | G4 | G5 | G3 ≤ | G3 ≤ (%) |
| Any | 6 | (37.5) | 1 | 3 | 1 | 1 | 0 | 2 | (12.5) |
| [Gastrointestinal disorders] | 1 | (6.3) | 0 | 1 | 0 | 0 | 0 | 0 | (0.0) |
| Periodontal disease | 1 | (6.3) | 0 | 1 | 0 | 0 | 0 | 0 | (0.0) |
| [General disorders and administration site conditions] | 1 | (6.3) | 0 | 1 | 0 | 0 | 0 | 0 | (0.0) |
| Pyrexia | 1 | (6.3) | 0 | 1 | 0 | 0 | 0 | 0 | (0.0) |
| [Infections and infestations] | 1 | (6.3) | 0 | 0 | 1 | 0 | 0 | 0 | (0.0) |
| bronchitis | 1 | (6.3) | 0 | 0 | 1 | 0 | 0 | 1 | (6.3) |
| Lung infection | 1 | (6.3) | 0 | 0 | 1 | 0 | 0 | 1 | (6.3) |
| [Investigations] | 1 | (6.3) | 0 | 0 | 0 | 1 | 0 | 1 | (6.3) |
| Neutrophil count decreased | 1 | (6.3) | 0 | 0 | 0 | 1 | 0 | 1 | (6.3) |
| [Respiratory, thoracic and mediastinal disorders | 2 | (12.5) | 1 | 1 | 0 | 0 | 0 | 0 | (0.0) |
| Dyspnea | 1 | (6.3) | 0 | 1 | 0 | 0 | 0 | 0 | (0.0) |
| Pleural effusion | 1 | (6.3) | 0 | 1 | 0 | 0 | 0 | 0 | (0.0) |
| Pneumonitis | 1 | (6.3) | 1 | 0 | 0 | 0 | 0 | 0 | (0.0) |

Abbreviations: TRAEs, treatment-related adverse events; SOC, system organ class; PT, preferred term; G, grade.

N is the number of patients in the safety analysis set. Data were presented as the number of patients. The percentage was calculated using N as the denominator. The adverse events recorded in the case report forms were coded, classified by SOC using MedDRA/J version 22.0, and were presented by PT.

Table S12. TEAEs leading to administration delay of docetaxel

| [SOC]<br>PT | Total, N = 16 | | Number of patients by worst grade | | | | | | |
|---|---|---|---|---|---|---|---|---|---|
| | n | (%) | G1 | G2 | G3 | G4 | G5 | G3 ≤ | G3 ≤ (%) |
| Any | 6 | (37.5) | 0 | 4 | 1 | 1 | 0 | 2 | (12.5) |
| [Gastrointestinal disorders] | 1 | (6.3) | 0 | 1 | 0 | 0 | 0 | 0 | (0.0) |
| Periodontal disease | 1 | (6.3) | 0 | 1 | 0 | 0 | 0 | 0 | (0.0) |
| [General disorders and administration site conditions] | 1 | (6.3) | 0 | 1 | 0 | 0 | 0 | 0 | (0.0) |
| Pyrexia | 1 | (6.3) | 0 | 1 | 0 | 0 | 0 | 0 | (0.0) |
| [Infections and infestations] | 2 | (12.5) | 0 | 1 | 1 | 0 | 0 | 0 | (0.0) |
| bronchitis | 1 | (6.3) | 0 | 1 | 0 | 0 | 0 | 0 | (0.0) |
| Upper respiratory tract infection | 1 | (6.3) | 0 | 0 | 1 | 0 | 0 | 1 | (6.3) |
| Lung infection | 1 | (6.3) | 0 | 0 | 1 | 0 | 0 | 1 | (6.3) |
| [Investigations] | 1 | (6.3) | 0 | 0 | 0 | 1 | 0 | 1 | (6.3) |
| Neutrophil count decreased | 1 | (6.3) | 0 | 0 | 0 | 1 | 0 | 1 | (6.3) |
| [Respiratory, thoracic and mediastinal disorders | 1 | (6.3) | 0 | 1 | 0 | 0 | 0 | 0 | (0.0) |
| Dyspnea | 1 | (6.3) | 0 | 1 | 0 | 0 | 0 | 0 | (0.0) |
| Pleural effusion | 1 | (6.3) | 0 | 1 | 0 | 0 | 0 | 0 | (0.0) |

Abbreviations: TEAEs, treatment-emergent adverse events; SOC, system organ class; PT, preferred term; G, grade.
N is the number of patients in the safety analysis set. Data were presented as the number of patients. The percentage was calculated using N as the denominator. The adverse events recorded in the case report forms were coded, classified by SOC using MedDRA/J version 22.0, and were presented by PT.

Table S13. TRAEs leading to administration delay of docetaxel

| [SOC] PT | Total, N = 16 n | (%) | Number of patients by worst grade G1 | G2 | G3 | G4 | G5 | G3 ≤ | G3 ≤ (%) |
|---|---|---|---|---|---|---|---|---|---|
| Any | 5 | (31.3) | 0 | 3 | 1 | 1 | 0 | 2 | (12.5) |
| [Gastrointestinal disorders] | 1 | (6.3) | 0 | 1 | 0 | 0 | 0 | 0 | (0.0) |
| Periodontal disease | 1 | (6.3) | 0 | 1 | 0 | 0 | 0 | 0 | (0.0) |
| [General disorders and administration site conditions] | 1 | (6.3) | 0 | 1 | 0 | 0 | 0 | 0 | (0.0) |
| Pyrexia | 1 | (6.3) | 0 | 1 | 0 | 0 | 0 | 0 | (0.0) |
| [Infections and infestations] | 1 | (6.3) | 0 | 0 | 1 | 0 | 0 | 1 | (6.3) |
| Bronchitis | 1 | (6.3) | 0 | 0 | 1 | 0 | 0 | 1 | (6.3) |
| Lung infection | 1 | (6.3) | 0 | 0 | 1 | 0 | 0 | 1 | (6.3) |
| [Investigations] | 1 | (6.3) | 0 | 0 | 0 | 1 | 0 | 1 | (6.3) |
| Neutrophil count decreased | 1 | (6.3) | 0 | 0 | 0 | 1 | 0 | 1 | (6.3) |
| [Respiratory, thoracic and mediastinal disorders | 1 | (6.3) | 0 | 1 | 0 | 0 | 0 | 0 | (0.0) |
| Dyspnea | 1 | (6.3) | 0 | 1 | 0 | 0 | 0 | 0 | (0.0) |
| Pleural effusion | 1 | (6.3) | 0 | 1 | 0 | 0 | 0 | 0 | (0.0) |

Abbreviations: TRAEs, treatment-related adverse events; SOC, system organ class; PT, preferred term; G, grade.
N is the number of patients in the safety analysis set. Data were presented as the number of patients. The percentage was calculated using N as the denominator. The adverse events recorded in the case report forms were coded, classified by SOC using MedDRA/J version 22.0, and were presented by PT.

Table S14. TEAE and TRAE leading to infusion interruption of trastuzumab

| [SOC]<br>PT | Total, N = 16 | | Number of Subjects by Worst Grade | | | | | | |
|---|---|---|---|---|---|---|---|---|---|
| | n | (%) | G1 | G2 | G3 | G4 | G5 | G3 ≤ | G3 ≤ (%) |
| Any | 1 | (6.3) | 0 | 1 | 0 | 0 | 0 | 0 | (0.0) |
| [Injury, poisoning and procedural complications] | 1 | (6.3) | 0 | 1 | 0 | 0 | 0 | 0 | (0.0) |
| Infusion related reaction | 1 | (6.3) | 0 | 1 | 0 | 0 | 0 | 0 | (0.0) |

Abbreviations: TEAEs, treatment-emergent adverse events; TRAEs, treatment-related adverse events; SOC, system organ class; PT, preferred term; G, grade. N is the number of patients in the safety analysis set. Data were presented as the number of patients. The percentage was calculated using N as the denominator. The adverse events recorded in the case report forms were coded, classified by SOC using MedDRA/J version 22.0, and were presented by PT.

Table S15. TEAEs and TRAEs leading to infusion interruption of docetaxel

| [SOC] | Total, N=16 | | Number of Subjects by Worst Grade | | | | | | |
|---|---|---|---|---|---|---|---|---|---|
| PT | n | (%) | G1 | G2 | G3 | G4 | G5 | G3 ≤ | G3 ≤ (%) |
| Any | 1 | (6.3) | 0 | 1 | 0 | 0 | 0 | 0 | (0.0) |
| [Cardiac disorders] | 1 | (6.3) | 1 | 0 | 0 | 0 | 0 | 0 | (0.0) |
| Palpitations | 1 | (6.3) | 1 | 0 | 0 | 0 | 0 | 0 | (0.0) |
| [Injury, poisoning and procedural complications] | 1 | (6.3) | 0 | 1 | 0 | 0 | 0 | 0 | (0.0) |
| Infusion related reaction | 1 | (6.3) | 0 | 1 | 0 | 0 | 0 | 0 | (0.0) |

Abbreviations: TEAEs, treatment-emergent adverse events; TRAEs, treatment-related adverse events; SOC, system organ class; PT, preferred term; G, grade. N is the number of patients in the safety analysis set. Data were presented as the number of patients. The percentage was calculated using N as the denominator. The adverse events recorded in the case report forms were coded, classified by SOC using MedDRA/J version 22.0, and were presented by PT.

Table S16. TEAEs and TRAEs leading to dose reduction of docetaxel

| [SOC] | Total, N = 16 | | Number of subjects by worst grade | | | | | | |
|---|---|---|---|---|---|---|---|---|---|
| PT | n | (%) | G1 | G2 | G3 | G4 | G5 | G3 ≤ | G3 ≤ (%) |
| Any | 8 | (50.0) | 0 | 3 | 3 | 2 | 0 | 5 | (31.3) |
| [Blood and lymphatic system disorders] | 2 | (12.5) | 0 | 0 | 2 | 0 | 0 | 2 | (12.5) |
| Febrile neutropenia | 2 | (12.5) | 0 | 0 | 2 | 0 | 0 | 2 | (12.5) |
| [General disorders and administration site conditions] | 2 | (12.5) | 0 | 2 | 0 | 0 | 0 | 0 | (0.0) |
| Pyrexia | 1 | (6.3) | 0 | 1 | 0 | 0 | 0 | 0 | (0.0) |
| Localized edema | 1 | (6.3) | 0 | 1 | 0 | 0 | 0 | 0 | (0.0) |
| [Infections and infestations] | 1 | (6.3) | 0 | 0 | 1 | 0 | 0 | 1 | (6.3) |
| Bronchitis | 1 | (6.3) | 0 | 0 | 1 | 0 | 0 | 1 | (6.3) |
| Lung infection | 1 | (6.3) | 0 | 0 | 1 | 0 | 0 | 1 | (6.3) |
| [Investigations] | 2 | (12.5) | 0 | 0 | 0 | 2 | 0 | 2 | (12.5) |
| Neutrophil count decreased | 2 | (12.5) | 0 | 0 | 0 | 2 | 0 | 2 | (12.5) |
| [Nervous system disorders] | 1 | (6.3) | 0 | 1 | 0 | 0 | 0 | 0 | (0.0) |
| Peripheral neuropathy | 1 | (6.3) | 0 | 1 | 0 | 0 | 0 | 0 | (0.0) |
| [Respiratory, thoracic and mediastinal disorders] | 2 | (12.5) | 0 | 2 | 0 | 0 | 0 | 0 | (0.0) |
| Pleural effusion | 2 | (12.5) | 0 | 2 | 0 | 0 | 0 | 0 | (0.0) |
| Dyspnea | 1 | (6.3) | 0 | 1 | 0 | 0 | 0 | 0 | (0.0) |

Abbreviations: TEAEs, treatment-emergent adverse events; TRAEs, treatment-related adverse events; SOC, system organ class; PT, preferred term; G, grade. N is the number of patients in the safety analysis set. Data were presented as the number of patients. The percentage was calculated using N as the denominator. The adverse events recorded in the case report forms were coded, classified by SOC using MedDRA/J version 22.0, and were presented by PT.
